# Separable and integrated pleasantness coding for appetitive and aversive odors across olfactory and ventral prefrontal cortices

**DOI:** 10.1038/s41467-026-73001-7

**Published:** 2026-05-22

**Authors:** Vivek Sagar, Christina M. Zelano, Thorsten Kahnt

**Affiliations:** 1 Department of Neurology, Feinberg School of Medicine, Northwestern University, Chicago, IL USA; 2 Present Address: Department of Psychological and Brain Sciences, Dartmouth College, Hanover, NH USA; 3 National Institute on Drug Abuse Intramural Research Program, Baltimore, MD USA

**Keywords:** Olfactory cortex, Cognitive neuroscience

## Abstract

Odor pleasantness is a key driver of approach and avoidance behaviors, raising the question of how pleasantness is represented in olfactory brain areas. To address this question, here we analyzed an existing dataset consisting of perceptual and fMRI responses to 160 odors from three individual participants. We find that piriform cortex, amygdala, orbitofrontal cortex, and ventromedial prefrontal cortex encode the pleasantness of appetitive and aversive odors. However, whereas these pleasantness representations are separable for appetitive and aversive odors in piriform cortex and amygdala, ventral prefrontal cortex (especially area 11) combines information from appetitive and aversive odors and forms a continuous representation of odor salience. These results suggest that distinct pleasantness codes for appetitive and aversive odors in olfactory cortices are integrated into a continuous representation in ventral prefrontal cortices.

## Abstract (web summary)

Odor pleasantness shapes approach and avoidance. Here, the authors show that the piriform cortex and amygdala encode the pleasantness of appetitive and aversive odors separately, while ventral prefrontal cortex represents a continuous salience code.

## Introduction

Pleasantness is a dominant perceptual feature of odors and directly tied to approach and avoidance responses^1^. As such, it is correlated with activity in olfactory brain areas, including piriform cortex, amygdala, and orbitofrontal cortex^2–12^.

Pleasantness is typically assumed to span a continuous dimension of valence from appetitive to aversive^9,13^. Despite our perceptual experience of a continuous dimension, given the vastly different repertoires of behavioral responses to appetitive and aversive stimuli^14^, the underlying neural coding scheme may not in fact be continuous. Indeed, work across species suggests that the brain may separately process the pleasantness of appetitive and aversive odors. Specifically, distinguishable coding of positive and negative odor valence has been reported in olfactory areas such as the olfactory bulb, olfactory tubercle, amygdala (AMY), piriform cortex (PirC), and elsewhere in the brain of flies^15^, rodents^15–17^ and humans^7,18,19^. If neural representations of odor pleasantness do not follow a continuous coding scheme in early olfactory areas, how does the brain achieve a continuous perceptual experience that may guide behavior?

**Fig. 1:**
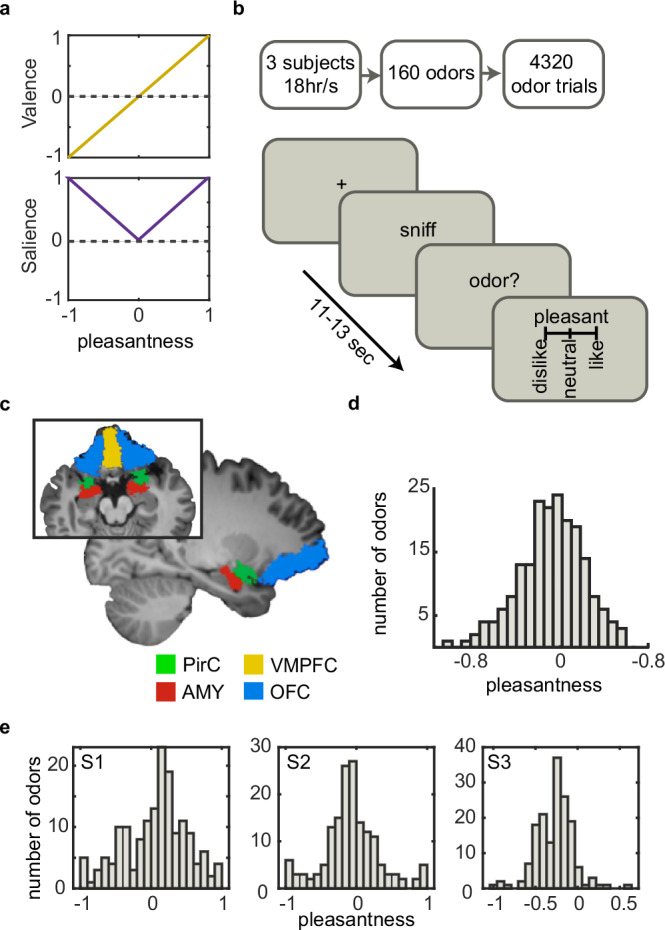
Experimental design and odor pleasantness ratings. **a** Odor valence (top) as a linear function of pleasantness, and salience (bottom) based on absolute pleasantness. **b** Experimental design. Three human participants were scanned for 18 h each (4320–4560 trials) as they smelled and rated odors on perceptual features including pleasantness. Odor pleasantness was rated on a labeled scale ranging from −10 = dislike very much, through 0 = neutral, to 10 = like very much. **c** Anatomical regions of interest (ROI) for participant S1: PirC piriform cortex, AMY amygdala, OFC orbitofrontal cortex, VMPFC ventromedial prefrontal cortex. **d** Histogram of pleasantness ratings across all odors and participants. **e** Histograms of pleasantness ratings across odors for individual participants.

Psychological theories often characterize affective experiences along continuous dimensions such as valence, intensity, salience and arousal^2,3,20,21^. Of note, different disciplines and studies define salience in different ways, emphasizing attention^22,23^, uncertainty^24,25^ or motivation and learning^26,27^. Here, we adopt a framework from behavioral and decision neuroscience, in which stimulus pleasantness (more generally, stimulus value) gives rise to two continuous dimensions, namely valence (Fig. 1a top) and salience (Fig. 1a bottom). Whereas valence is based directly on pleasantness and thought to drive approach-avoidance responses^9,13^, salience is defined as absolute pleasantness (very pleasant or very unpleasant odors have high salience) and relates to the magnitude of the affective response^28–31^.

Human studies in other sensory modalities suggest that the brain represents such continuous dimensions of valence and salience in cortical areas^28,32,33^. For instance, activity in ventromedial prefrontal cortex (VMPFC) correlates with the value of both appetitive and aversive stimuli^34^, and response patterns in orbitofrontal cortex (OFC) represent value across cues that predict positive and negative monetary outcomes^28^. While this implies ventral prefrontal regions as key candidates for integrating pleasantness information across valence domains into continuous dimensions, studies in rodents^29,30,35^ and humans^9^ have shown similar signals in subcortical structures.

Here, we hypothesized that olfactory cortices employ separate coding schemes for appetitive and aversive odors, which are subsequently integrated into continuous dimensions of valence and salience in ventral prefrontal cortices. To test these ideas, we leveraged an existing high-precision olfactory functional magnetic resonance imaging (fMRI) dataset consisting of 18 h of fMRI responses to 160 odors per participant from three human participants^36^. First, we used representational similarity and decoding analyses to test whether distributed activity patterns in early olfactory areas (PirC and AMY) and ventral prefrontal cortices (OFC and VMPFC) represent the valence and salience of odors across a wide range of pleasantness. Finding robust representations of both valence and salience, we next applied decoding analyses to test for encoding of pleasantness separately within appetitive (pleasant) and aversive (unpleasant) odors. This revealed that the pleasantness of appetitive and aversive odors is represented in each of these regions. Finally, we used a cross-decoding approach to examine whether pleasantness codes generalize across appetitive and aversive odors, forming continuous representations of valence and/or salience. We find that only OFC (area 11) represents a common dimension of salience that integrates across both aversive and appetitive odors. Together, our findings indicate that the pleasantness of appetitive and aversive odors is represented via separable coding schemes in olfactory areas, whereas ventral prefrontal cortex represents an integrated dimension of odor salience.

## Results

### Odor pleasantness, valence, and salience

The current analyses are based on previously published perceptual and functional magnetic resonance imaging (fMRI) data from three human participants in response to 160 monomolecular odors (Fig. 1b, Supplementary Table 1)^36^. We focused our analyses on brain regions involved in olfactory function and hedonic processing: Piriform cortex (PirC), amygdala (AMY), and orbitofrontal cortex (OFC) and ventromedial prefrontal cortex (VMPFC) (Fig. 1c).

On each trial, participants smelled one of 160 monomolecular odors and rated it on one of several perceptual descriptors, including pleasantness (scale ranging from −10 = dislike very much, through 0 = neutral, to +10 = like very much, rescaled to −1 to 1 for all subsequent analyses). Roughly half of the odors were appetitive (pleasantness rating >0: 41.54%) and aversive (pleasantness rating <0: 58.46%, Fig. 1d, e, Supplementary Table 1).

Two continuous dimensions can be derived from these pleasantness ratings. First, valence (signed pleasantness, Fig. 1a top) indicates the degree to which an odor is pleasant or liked, from negative valence for aversive odors to positive valence for appetitive odors. Second, salience (absolute pleasantness, Fig. 1a bottom) indicates the degree to which an odor is affectively important regardless of whether it is appetitive or aversive, describing a u-shaped function of pleasantness. Of note, neural coding of continuous dimensions of valence and salience cannot be distinguished within a stimulus set consisting exclusively of appetitive or aversive odors but requires neural responses to both types of odors.

### Neural representations of odor valence and salience

**Fig. 2:**
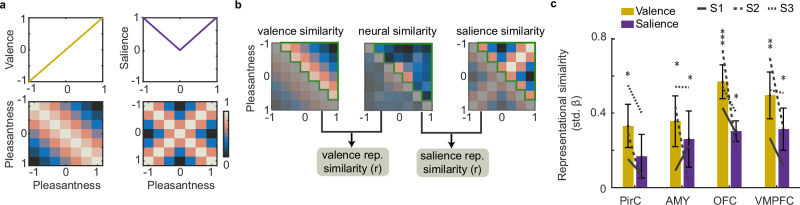
Neural representations of odor valence and salience. **a** (Top) Valence and salience as a function of pleasantness. (Bottom) Hypothetical representational similarity matrices (RSM) for odor valence and salience based on binned pleasantness ratings. **b** Representational similarity analysis. For each participant and brain region, we computed the similarity between the voxel responses for all bin pairs with a given pleasantness rating (averaged across *n* = 200 trials with the same pleasantness ratings). Next, we computed the correlation between this neural RSM with the valence and salience RSM. **c** Mean standardized regression coefficients (beta estimates) for valence and salience from a linear model predicting the neural RSM across *n* = 3 participants. For each participant, coefficients were estimated from the 21 off-diagonal elements of the 7×7 neural RSM. The coefficients for valence and salience are significant in all regions except salience for PirC. (valence: PirC, β = 0.331, *p* = 0.027; AMY, β = 0.356, *p* = 0.031; OFC, β = 0.569, *p* < 0.001; VMPFC, β = 0.495, *p* = 0.001; salience: PirC, β = 0.169, *p* = 0.182; AMY, β = 0.260, *p* = 0.046; OFC, β = 0.303, *p* = 0.026; VMPFC, β = 0.314, *p* = 0.019; one-tailed permutation test, 10,000 permutations). Data are presented as mean values and error bars indicate standard error of the mean. Asterisks denote *p < 0.05, **p < 0.01 and ***p < 0.001, respectively.

As a first step, we used Representational Similarity Analysis (RSA) to test if valence and salience are represented in our regions of interest (ROI). We sorted pleasantness ratings into seven equally spaced bins and created hypothetical representational similarity matrices (RSM) for valence and salience (Fig. 2a). These matrices capture the similarity in valence and salience between all pairs of pleasantness bins. Next, we constructed the corresponding neural RSM in each ROI and participant by correlating multi-voxel pattern responses between pairs of pleasantness bins (Supplementary Fig. 1). We then examined the overlap between the off-diagonal elements of the neural and hypothetical RSMs for valence and salience (Fig. 2b). Specifically, we simultaneously regressed the valence and salience RSM against the neural RSM and obtained regression coefficients (β) as measures of valence and salience coding in each brain region. In line with previous studies, valence and salience were significantly represented in all olfactory areas as well as OFC and VMPFC (all *p*’s <0.05, except salience in PirC, one-tailed permutation test, for exact *p*-values see legend Fig. 2c).

Of note, these results were robust to sniffing-related information (Supplementary Fig. 2), different bin sizes (Supplementary Fig. 3), and significant in other cortical areas but not in a white-matter ROI (Supplementary Fig. 4). Further, replacing pleasantness with other perceptual descriptors such as intensity, warm, edibility or familiarity did not yield comparable results (Supplementary Fig. 5).

**Fig. 3:**
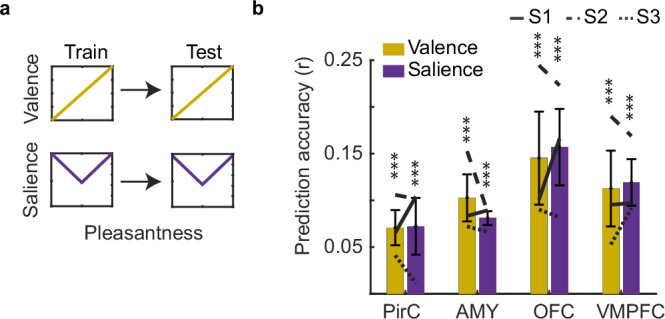
Decoding odor valence and salience. **a** Schematic showing the Support vector regression (SVR) analysis to predict valence (or salience) from voxel response patterns. **b** Mean prediction accuracy (Pearson’s correlation between predicted and actual valence/salience in held out test sets) is significant in all brain regions across *n* = 3 participants. (valence: PirC, *r* = 0.071, *p* < 0.001; AMY, *r* = 0.103, *p* < 0.001; OFC, *r* = 0.145, *p* < 0.001; VMPFC, *r* = 0.113, *p* < 0.001; salience: PirC, *r* = 0.072, *p* < 0.001; AMY, *r* = 0.081, *p* < 0.001; OFC, *r* = 0.157, *p* < 0.001; VMPFC, *r* = 0.119, *p* < 0.001; *N* = 4400 trials; two-tailed *t*-test). Data are presented as mean values and error bars indicate standard error of the mean. Asterisks denote *p < 0.05, **p < 0.01 and ***p < 0.001, respectively.

To confirm these findings on a trial-by-trial level, we performed a decoding analysis to test if valence and salience can be decoded from neural activity patterns in these areas. Specifically, we used cross-validated support vector regression (SVR) models to predict trial-wise odor valence or salience from fMRI activity patterns in each brain region and participant (Fig. 3a). Prediction accuracy (Pearson correlation between predicted and actual valence/salience) was significant in all regions (all *p*’s <0.001, two-tailed *t*-test, for exact *p*-values see legend Fig. 3b), indicating that valence and salience can be decoded from single-trial activity patterns in these regions.

### Pleasantness coding for appetitive and aversive odors

The previous analyses tested valence and salience coding across the entire range of pleasantness, presupposing that neural coding of pleasantness generalizes across appetitive and aversive odors, which is not necessarily the case. Moreover, results from these analyses could have been driven by categorical differences between appetitive and aversive odors rather than continuous levels of valence. Thus, we next performed a decoding analysis to examine and compare pleasantness coding for appetitive and aversive odors.

**Fig. 4:**
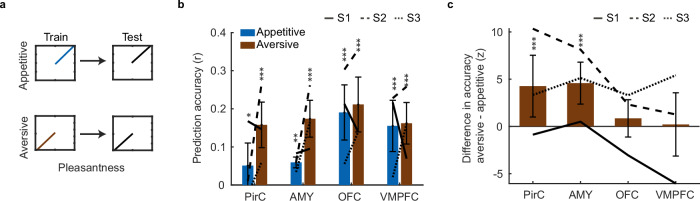
Pleasantness coding for appetitive and aversive odors. **a** SVR model to predict pleasantness of appetitive (or aversive) odors from appetitive (or aversive) odor trials. **b** Mean prediction accuracy (Pearson’s correlation between predicted and actual pleasantness in held out test sets) across *n* = 3 participants is significant in all brain regions. (Appetitive valence: PirC, *r* = 0.051, *p* = 0.017; AMY, *r* = 0.059, *p* = 0.006; OFC, *r* = 0.190, *p* < 0.001; VMPFC, *r* = 0.155, *p* < 0.001; *N* = 2168 trials; Aversive valence: PirC, *r* = 0.158, *p* < 0.001; AMY, *r* = 0.174, *p* < 0.001; OFC, *r* = 0.211, *p* < 0.001; VMPFC, *r* = 0.162, *p* < 0.001; *N* = 2177 trials; two-tailed *t*-test) **c** Difference in prediction accuracies for aversive and appetitive odors (z transformed values) with positive values indicating better decoding for aversive odors, averaged across *n* = 3 participants. The difference is significant in PirC and AMY with higher prediction accuracies for aversive odors (PirC, *z* = 4.263, *p* < 0.001; AMY, *z* = 4.584, *p* < 0.001; OFC, *z* = 0.850, *p* = 0.160; VMPFC, *z* = 0.209, *p* = 0.804; two-tailed Steiger’s z-test, uncorrected). Data are presented as mean values and error bars indicate standard error of the mean. Asterisks denote *p < 0.05, **p < 0.01 and ***p < 0.001, respectively.

Specifically, we used SVR models to predict the pleasantness of appetitive odors when training exclusively on data from appetitive odors (Fig. 4a), and to predict the pleasantness of aversive odors when training exclusively on data from aversive odors. We obtained significant prediction accuracy (Pearson’s correlation between predicted and actual pleasantness) for the pleasantness of both appetitive and aversive odors in PirC, amygdala, as well as OFC and VMPFC (all *p*’s <0.05; two-tailed *t*-test, for exact *p*-values see legend Fig. 4b). However, in PirC and amygdala, prediction accuracies for aversive odors were significantly higher than those for appetitive odors (*p* < 0.05, for exact *p*-values see legend Fig. 4c). While these results indicate widespread pleasantness coding for both appetitive and aversive odors, they suggest that pleasantness coding for aversive and appetitive odor may differ in early olfactory areas.

Of note, we obtained similar results when accounting for differences in the number of appetitive vs. aversive odors, the number of voxels in each ROI, and odor intensity (Supplementary Fig. 6). We did not find significant pleasantness coding for appetitive and aversive odors using a voxel-wise univariate analysis (Supplementary Fig. 7).

### Integrated coding of salience in ventral prefrontal cortex

**Fig. 5:**
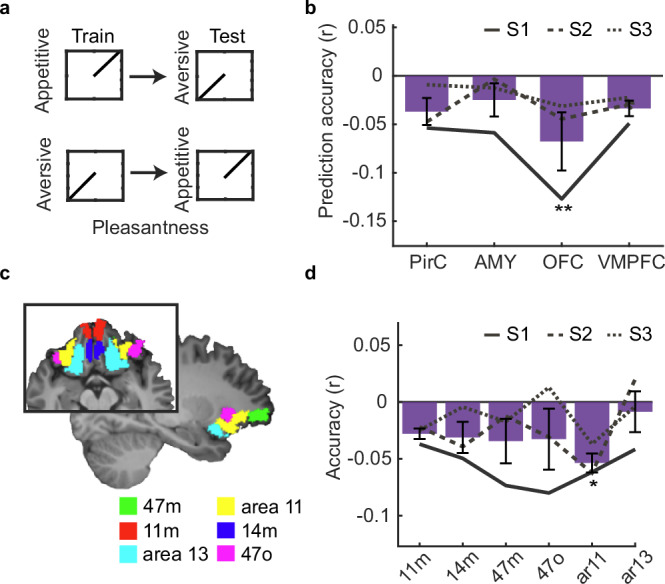
Integrated coding of salience in ventral prefrontal cortex. **a** Schematic of SVR model to predict pleasantness of appetitive (or aversive) odors when trained on aversive (or appetitive) odor trials. **b** Mean decoding accuracy across *n* = 3 participants is not significantly above chance in any brain region but is significantly below chance in OFC (PirC, *r* = −0.037, *p* = 0.084; AMY, *r* = −0.025, *p* = 0.242; OFC, *r* = −0.068, *p* = 0.002; VMPFC, *r* = −0.034, *p* = 0.115; *N* = 2197 trials, two-tailed *t*-test). **c** Anatomical subregions of interest (ROI) in ventral prefrontal cortex for participant S1: **d** Mean decoding accuracy across *n* = 3 participants is not significantly above chance in any subregion but is significantly below chance in area 11 (11 m, *r* = −0.0279, *p* = 0.191; 14 m, *r* = −0.031, *p* = 0.145; 47 m, *r* = −0.034, *p* = 0.107; 47o, *r* = −0.033, *p* = 0.125; ar11, *r* = −0.054, *p* = 0.012; ar13, *r* = −0.009, *p* = 0.692, *N* = 2197 trials, two-tailed *t*-test). Data are presented as mean values and error bars indicate standard error of the mean. Asterisks denote *p < 0.05, **p < 0.01 and ***p < 0.001, respectively.

The previous analyses suggest that both olfactory and ventral prefrontal cortices code the pleasantness of appetitive and aversive odors. However, these analyses do not reveal whether the pleasantness of appetitive and aversive odors is represented independently or whether they are integrated into common representations of value or salience. To test this, we conducted a trial-wise cross-decoding analysis, where an SVR was used to predict the pleasantness of appetitive (or aversive) odors after being trained on activity patterns from aversive (or appetitive) odors (Fig. 5a).

Significant above-chance prediction accuracy for the pleasantness of appetitive odors from a model trained exclusively on aversive odors (aversive→appetitive, and vice-versa appetitive→aversive) would indicate that an area represents the valence of both appetitive and aversive odors using the same continuous neural code for valence. In contrast, significant below-chance prediction accuracy (highly aversive odors are classified together with highly appetitive odors, and vice versa) would indicate that an area represents the salience of aversive and appetitive odors using the same neural code for salience. Finally, cross-decoding prediction accuracy around chance in areas that code the pleasantness of aversive and appetitive odors would suggest that these areas represent the pleasantness of appetitive and aversive odors using independent neural codes.

Cross-decoding prediction accuracy (averaged for appetitive→aversive and vice-versa) was not significantly different from chance in PirC and amygdala (*p* > 0.05; two tailed *t*-test, for exact *p*-values see legend Fig. 5b), suggesting separable pleasantness coding for appetitive and aversive odors in these areas. In contrast, prediction accuracy was significantly below chance in the OFC (*p* < 0.05; for exact p-values see legend Fig. 5b), suggesting that OFC may represent an integrated code for odor salience.

The ventral prefrontal cortex is a large and heterogeneous brain area that can be subdivided based on anatomical features^37–39^. To examine which subregions show integrated pleasantness coding, we repeated the cross-decoding analysis in subregions 11 m, 14 m, 47 m, 47o, area 11, and area 13 from the Neubert et al. atlas^37^. Although several areas showed nominal below-chance prediction accuracy, cross-decoding accuracy was only significantly below chance (indicating salience coding) in area 11 (Fig. 5c).

Taken together, these results indicate that OFC, especially area 11, integrates the distinct pleasantness signals for appetitive and aversive odors into a continuous representation of salience.

## Discussion

In this study, we used high-precision imaging data to examine how the human brain represents the pleasantness of odors. We find that olfactory cortices contain independent representations for the pleasantness of appetitive and aversive odors, whereas areas in the ventral prefrontal cortex integrate across appetitive and aversive odors to form a continuous representation of salience.

Our results revealed that neural representations of pleasantness for appetitive and aversive stimuli show little overlap in PirC and amygdala. Although the pleasantness of appetitive and aversive odors was robustly represented in both areas, a decoder trained on aversive stimuli was unable to predict the pleasantness of appetitive stimuli, and vice versa. This suggests a valence-based separation at an early stage of olfactory processing, as has been suggested by previous work^15,18^. Given that odor pleasantness can be predicted from the chemical structure of the odorous molecule itself^1,40^, this separation may directly result from the glomerular organization of the olfactory bulb, where sensory neurons expressing the same receptor gene converge on the same glomeruli^41–43^. This may be particularly true for the amygdala, which is known to conserve some of the direct glomeruli-specific projections from the bulb^44^, especially for orchestrating innate behavioral responses to odors^45,46^. While several studies have examined odor valence coding in humans and animals, parallels between species need to be inferred. Work in humans suggests that odor pleasantness is transmitted from the olfactory bulb to the PirC^18^, but given the diffuse and overlapping input into PirC^47,48^, as well as its general association cortex-like architecture^49,50^ as known from rodent studies, the valence-based segregation in PirC may also be generated locally or imposed by top down input.

We considered olfactory valence and salience as two candidate dimensions which could integrate across the distinct pleasantness representations. While valence (or value) is linearly related to pleasantness, salience has been defined in numerous ways across studies and disciplines^27^. For example, in vision sciences, salience or saliency relates to physical stimulus attributes that elicit attention^22,23^, whereas definitions in the associative learning field focus on uncertainty and predictiveness^24,25^. Our definition is related to motivational salience in the behavioral neuroscience and neuroeconomics literature^26,31^, operationalizing salience as the absolute value of pleasantness^28–30^. However, we do acknowledge that this formalization does not incorporate other potentially salience-related stimulus dimensions such as familiarity, intensity, arousal etc., which require additional ratings or behavioral assessments to be captured appropriately.

Our findings mirror previous results demonstrating odor valence coding in the human amygdala^3,10^. However, the current demonstration of separable pleasantness coding in this area fundamentally extends this prior work, and may even appear to conflict with previous findings suggesting continuous valence coding in the amygdala^9^. However, we observed similar evidence for continuous valence coding in the amygdala when using analysis methods that presuppose such coding schemes (Figs. 2c and 3b), which may have been driven by categorical differences between appetitive and aversive odors. Non-overlapping pleasantness coding for appetitive and aversive odors in the amygdala was only revealed by applying within-participant cross-decoding approaches (Fig. 5a), highlighting the strength or our precision imaging approach. Moreover, this notion of discontinuous pleasantness coding parallels previous work showing that pleasure and displeasure evoked by visual stimuli do not fall on a unidimensional axis^51^.

Like PirC and amygdala, ventral prefrontal areas showed robust representations of valence and salience when collapsing across appetite and aversive odors (Figs. 2c and 3b). These areas also robustly represented the pleasantness of appetitive and aversive odors when considered in isolation (Fig. 4b). However, only in the OFC, especially area 11, did pleasantness-coding activity patterns generalize across aversive and appetitive odors, forming an integrated representation of salience. Surprisingly, this cross-decoding approach did not reveal any evidence for an integrated representation of odor valence. This contrasts with findings from decision-making studies in humans and non-human primates, showing overlapping value codes for appetitive and aversive outcomes in ventral prefrontal cortex^28,34,52^. This discrepancy may suggest a unique coding scheme for odor pleasantness in ventral prefrontal areas that primarily represents the affective salience of odors, independent of whether they are appetitive or aversive.

We also found valence and salience-related signals outside our primary regions of interest, in line with previous work^10,53^ suggesting that affective information is widely distributed across the brain^54^. Of note, our analyses examined neural representations of pleasantness in multi-voxel activity patterns. The size of a fMRI voxel almost certainly exceeds the size of neuronal populations with homogenous coding schemes. Thus, the information sampled by our approach likely results from a combination of factors, including clustering of neuronal populations with similar tuning, biased sampling of neuronal populations at level of individual voxels^55,56^, and filtering properties of the vasculature^57,58^.

In summary, our findings reveal that whereas processing of pleasantness for aversive and appetitive odors is separate in early olfactory areas, ventral prefrontal cortices integrate across domains and represent a continuous dimension of salience. The synthesis of a continuous dimension from separate representations offers flexibility in the shape of this integration; As contextual demands may change how aversive and appetitive stimuli are best interpreted to maximize reward and minimize punishment^59^, flexible integration may serve adaptive behavior. Thus, our findings exemplify a mechanism by which the brain extracts biologically relevant information from discrete stimuli (odorous molecules) and how prefrontal cortex integrates this information to support perception and behavior.

## Methods

### Dataset

The analyses were based on a previously published fMRI dataset where 3 healthy human participants (two females aged 23–24 years, one male aged 24 years based on self-reports) participated in an olfactory task involving smelling and rating 160 odors (Supplementary Table 1) inside the scanner^36^. This study was approved by the Institutional Review Board (IRB) at Northwestern University and adhered to the Declaration of Helsinki and the Belmont Report. Participation was voluntary and written informed consent was obtained from all participants. All participants were compensated for their participation consistent with IRB-approved protocols. We did not carry out sex-based analysis because of the small n and the fact that sex was unlikely to influence our main findings. No statistical method was used to predetermine sample size. No data was excluded from the previously published dataset. Original dataset removed one additional subject prior to analysis due to a psychiatric disorder that was disclosed after data collection. The order of the stimuli was randomized across experimental sessions. The Investigators were not blinded to allocation during experiments and outcome assessment

Each participant underwent extensive scanning sessions totaling ~18 h, resulting in over 4300 trials per participant (S1: 4560 trials, S2 and S3: 4320 trials). On each trial, participants were presented with one of the odors and rated it on several perceptual descriptors, including pleasantness. Descriptor ratings were provided on a scale from −10 to +10, which were rescaled by dividing them by 10 (range −1 to +1) for subsequent analyses.

The pleasantness of each odor was rated at least 3 times. For S1 all three ratings were collected inside the scanner, while for S2 and S3 two ratings were acquired outside the scanner. The pleasantness ratings were correlated across sessions in each participant (Pearson’s r: S1 = 0.479, S2 = 0.668, S3 = 0.551, *p* < 0.001, two-tailed *t*-test).

Approximately 41.54% of the odors were rated as appetitive (pleasantness rating >0) and 58.46% as aversive (pleasantness rating <0, Supplementary Table 1). Participants S2 and S3 also performed additional ratings outside the scanner using a self-paced behavioral task. We measured breathing using a breathing belt (S1) or a pneumotachometer and spirometer (S2 and S3).

We acquired fMRI data at a 3 T PRISMA system (Siemens) using gradient echo T2*-weighted EPI sequence. Images were acquired at a TR of 1.4 s and spatial resolution of 2 mm isotropic for S1 and 1.7 mm in plane and 2 mm slice thickness for S2 and S3. Following standard preprocessing steps such as realignment, coregistration and spatial smoothing, we used Friston-24 as nuisance regressors^60^. We appended additional nuisance regressors such as sniff flow, breathing volume, slice differences and variance (to account for within-volume motion). We then used the GLM single package^61,62^ that fits custom HRFs for each voxel in addition to denoising and fractional ridge regression to extract trial-wise voxel responses.

### Regions of interest (ROI)

We focused on a set of olfactory and ventral prefrontal regions of interest (ROI), namely the piriform cortex (PirC), amygdala (AMY), orbitofrontal cortex (OFC), and ventromedial prefrontal cortex (VMPFC). We obtained the olfactory ROI masks from our previously published dataset^36,63^ and the VMPFC mask from the AAL atlas. All masks were inverse normalized to each participant’s native space using their T1 images, with manual hand drawn corrections. All analyses were based on gray matter voxels in each ROI only. Details about the experimental setup, perceptual descriptors, task design and scanning parameters have been reported in a previous study^36^. However, unlike our previous study, analysis was based on all gray-matter voxels within each ROI. We also performed additional analyses in the insula, hippocampus, auditory cortex, and a white matter control region defined similarly as in our previous study using this dataset^36^.

### Representational similarity analyses (RSA)

We used Representational Similarity Analysis (RSA)^64^ to investigate how valence and salience are represented in neural activity patterns within the ROIs. For each participant, pleasantness and absolute pleasantness ratings were sorted into seven equally spaced bins and trials were assigned to the bins based on the pleasantness of the odor delivered. We subsampled trials to ensure there were equal number of trials in each pleasantness bin (number of trials = 200). This ensured that the results were not biased by higher sampling of bins with neutral odors, nor because of an asymmetry in the number of appetitive and aversive odors. We then created bin-by-bin neural representational similarity matrices in each ROI and participant based on Pearson’s correlation of voxel responses for a pair of bins. We then made hypothetical representational similarity matrices (RSMs) for both valence and salience based on our definition of valence as linear function of pleasantness and that of salience as absolute pleasantness. To assess the degree to which activity patterns reflected valence and salience, the off-diagonal elements of the neural RSMs were regressed onto the corresponding elements of the hypothetical valence and salience RSMs using a linear regression model. We used standardized regression coefficients (β) as a measure of representation of valence and salience in the multivoxel pattern for each ROI. We repeated the subsampling process 1000 times and averaged the regression coefficient obtained across all iterations.

Here we performed the analysis based on discretizing pleasantness in 7 bins. We chose 7 bins since it optimized the trade-off between a relatively high resolution and a sufficient numbers of trials per bin.

We adapted a univariate RSA to examine coding of pleasantness for appetitive and aversive odors in individual voxels. In other words, for each voxel we created a bin-by-bin neural similarity matrix by taking pairwise absolute differences in voxel responses for two bins of pleasantness (each consisting of the same number of odor trials). Then, similar to the multivariate RSA, we regressed hypothetical similarity matrices for appetitive and aversive pleasantness against the neural RSM for every voxel, resulting in a bivariate estimate of voxel-wise representation of pleasantness for appetitive and aversive odors.

We used permutation tests to determine statistical significance, where for each of the 10,000 permutations, the cells of the neural response matrix (shaped number of voxels × number of bins) were randomly shuffled for each voxel. We then repeated the RSA with the shuffled neural response matrix to generate a null distribution. *P*-values were calculated based on the proportion of permuted coefficients exceeding the observed β. We also performed RSA in other brain areas, including a white matter control region. We did not obtain a significant effect in the white matter (Supplementary Fig. 5). These permutation analyses were performed at the level of individual subjects. For each participant and each region, we therefore obtained a null distribution of regression coefficients. Similar to our previous work^36^ we averaged the regression coefficients across participants and performed group-level statistical analysis from the average null distribution across participants. Since RSA coefficients cannot be meaningfully negative, we performed a one-tailed test and obtained a *p*-value of the representational similarities based on 1 - percentile values in the null distribution. All statistical analyses were based on *n* = 3 participants that served as biological replicates.

### Basic decoding analyses

We used support vector regression (SVR) models to predict trial-by-trial valence and/or salience ratings from neural activity patterns as model features. Separate SVR models were trained for valence and salience within each ROI and participant. We used split half cross-validation for more optimal regularization of cross-validation. Prediction accuracy was quantified as the Pearson correlation coefficient between the predicted and actual pleasantness ratings across test trials in the held-out data. We performed a similar decoding analysis separately for appetitive and aversive odors.

Since permutation tests were not computationally feasible for our single-trial decoding analyses, we performed participant-wise parametric tests for each ROI. Specifically, we computed the *p*-value for participant-wise prediction accuracies (correlation coefficients) using two-tailed *t*-tests, with the degrees of freedom corresponding to the number of predictions (i.e., trials) per participant. These results are summarized in Supplementary Tables 2, 3. For the purposes of summarizing and illustration, we also performed group-level analyses where we computed the *p*-value of the average Fisher’s Z-transformed correlation coefficients across participants using a two-tailed *t*-test with the degrees of freedom corresponding to the mean number of predictions (i.e., trials) across participants. The values reported in the figures were inverse-transformed to reflect raw correlations.

For both kinds of decoding analyses (valence/salience and appetitive/aversive pleasantness), we performed additional control analyses (as described in Supplementary Fig. 6). As a first control, we accounted for non-uniformity in the histogram of pleasantness values. We accounted for imbalance in the number of appetitive vs. aversive odors by randomly choosing an equal number of trials (500) for appetitive and aversive odors. We then used the SVR to decode pleasantness values on the reduced dataset with equal number of trials per bin. We averaged the decoding results twenty times for numerical robustness. As a second control, we accounted for differences in voxel size across ROI by performing decoding using a fixed number of voxels (100 across all ROI). Lastly, we controlled for intensity, by regressing it out from pleasantness values prior to discretization. For all decoding analyses, we z-scored the training data across trials for each voxel and used the mean and standard deviation of the training data to normalize the trials in the test data set.

For all decoding analyses, we chose the LibSVM SVR with default parameters (cost = 1, nu = 0.5, gamma = 1/num features) with radial kernel and split-half for cross validation.

### Cross-decoding analyses

To test whether pleasantness codes for appetitive and aversive odors are integrated into a continuous, and whether this dimension reflects valence or salience, we performed a cross-decoding analysis. Namely, we trained an SVR on pleasantness ratings of appetitive odors and used the trained model to predict the pleasantness of aversive odors, and vice versa. Prediction accuracies (Pearson correlation coefficient) were Fisher’s Z transformed and averaged across the two models (i.e., train on appetitive, test on aversive and train on aversive, test on appetitive odors).

We ran these analyses for each of our primary ROIs as well as on the Neubert et al. subdivisions of ventral prefrontal cortex^37^. For this we inverse-normalized areas: 11 m, 14 m, 47 m, 47o, area 11, and area 13 from MNI space to participant’s native spaces.

### Reporting summary

Further information on research design is available in the Nature Portfolio Reporting Summary linked to this article.

## Supplementary information

Supplemental information

Reporting Summary

Transparent Peer Review file

## Data Availability

Dataset to reproduce all major findings of the study is available without restriction at https://github.com/viveksgr/ARC and archived on Zenodo 10.5281/zenodo.19119376^65^. Analyses presented in this manuscript are based on previously published dataset 10.5281/zenodo.7636722^63^. The access request to the raw dataset can be submitted at Zenodo and is subject to a data-use agreement that restricts use to research purposes, prohibits re-identification and redistribution, and requires citation of the associated Zenodo DOI and publication. The timeframe for response to requests is approximately 10 business days.

## Code availability

All analyses were performed using custom scripts in MATLAB 2023b and the LibSVM package^66^ for decoding. Other software packages used in the study were SPM12^67^, GLMSingle^61,62^, and Breathmetrics toolbox^68^. Code for preprocessing and reproducing the results presented in this manuscript is available at https://github.com/viveksgr/ARC and archived on Zenodo 10.5281/zenodo.19119376^65^.
