# Supplementary material for: Separable and integrated pleasantness coding for appetitive and aversive odors across olfactory and ventral prefrontal cortices
Source: Nat Commun. 2026 May 22;17:6732. doi: 10.1038/s41467-026-73001-7 (PMC13385377; doi:10.1038/s41467-026-73001-7)
Supplement: Supplementary file 1 — Supplemental information [file 41467_2026_73001_MOESM1_ESM.pdf]

**SUPPLEMENTARY INFORMATION**

# Separable and integrated pleasantness coding for appetitive and aversive odors across olfactory and ventral prefrontal cortices

Vivek Sagar[1,3], Christina M. Zelano[1], Thorsten Kahnt[2*]

[1]Department of Neurology, Feinberg School of Medicine, Northwestern University, Chicago, IL, 60611, USA

[2]National Institute on Drug Abuse Intramural Research Program, Baltimore, MD, 21224, USA

[3]Present address: Department of Psychological and Brain Sciences, Dartmouth College, Hanover, NH, 03755, US

[*]Corresponding author: thorsten.kahnt@nih.gov

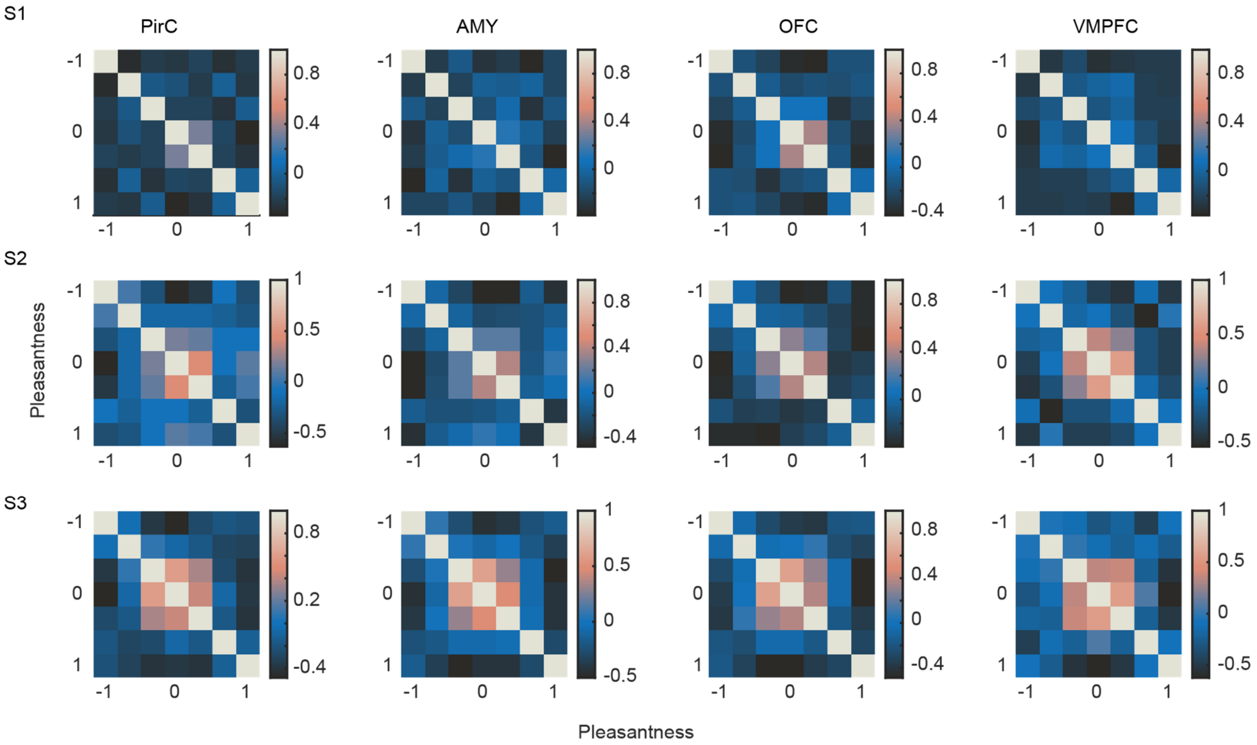


**Supplementary Figure 1.** Representational similarity matrices for different brain areas and participants

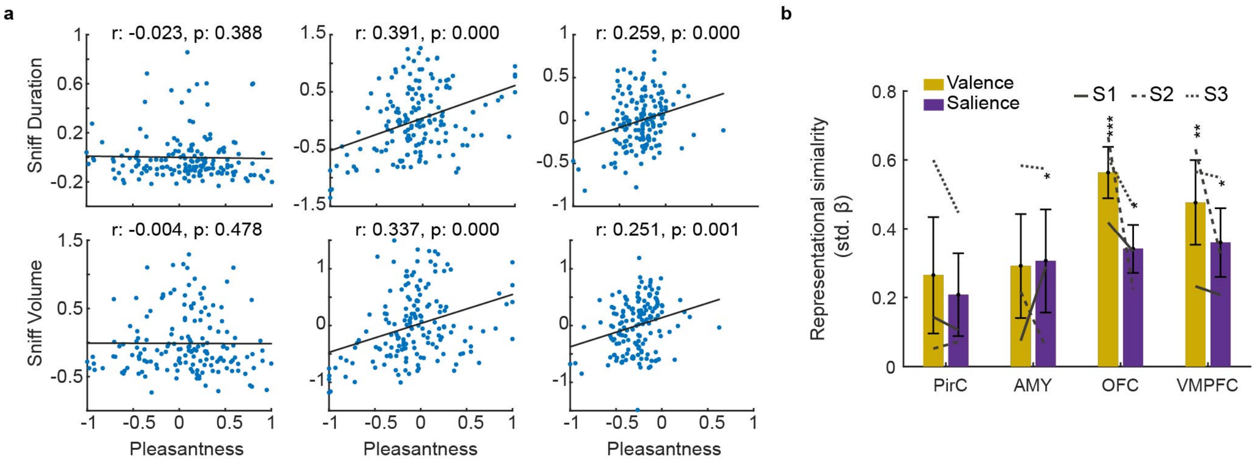


**Supplementary Figure 2.** Control representational similarity analysis to account for sniffing related effects. **a**. (Top) Sniff duration plotted as a function of pleasantness for 3 participants. Data points correspond to odors (averaged across trials). Sniff Duration is positively correlated with pleasantness in S2 and S3 but not S1. **a**. (Bottom) Sniff volume plotted as a function of pleasantness for 3 participants. Data points correspond to odors (averaged across trials). Sniff volume is positively correlated with pleasantness in S2 and S3 but not S1. **b**. Representational similarity analysis controlling for sniff related effects. A bin-by-bin sniff correlation matrix was constructed and added as a nuisance regressor in the linear model. Effect of valence is significant in OFC and VMPFC, and salience is significant in AMY, OFC and VMPFC across n = 3 participants (valence: PirC, β=0.268, p=0.170; AMY, β=0.291, p=0.124; OFC, β=0.564, p<0.001; VMPFC, β=0.480, p=0.003; salience: PirC, β=0.206, p=0.171; AMY, β=0.309, p=0.039; OFC, β=0.339, p=0.019; VMPFC, β=0.361, p=0.007; one-tailed permutation test). Data are presented as mean values and error bars indicate standard error of the mean. Asterisks denote *, p<0.05, **, p<0.01 and ***, p<0.001, respectively.

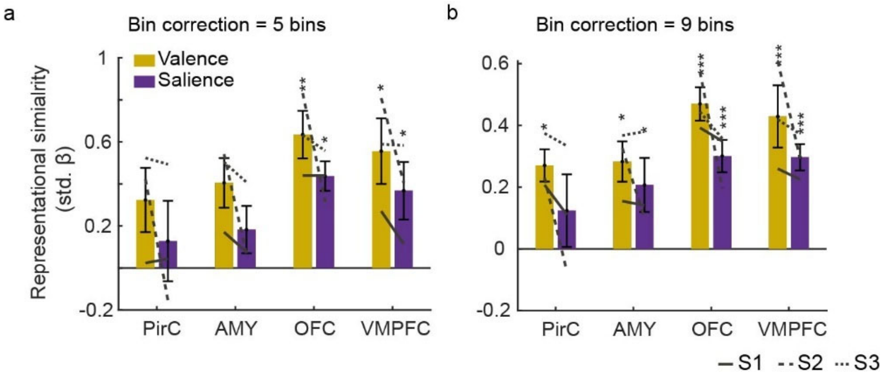


**Supplementary Figure 3:** Representational similarity analysis controlling for number of bins used for discretizing pleasantness. **a.** RSA when pleasantness ratings are discretized with 5 bins. The coefficients for valence and salience are significant in OFC and VMPFC and trending for valence in AMY across n=3 participants (valence: PirC, β=0.328, p=0.141; AMY, β=0.409, p=0.063; OFC, β=0.635, p=0.003; VMPFC, β=0.561, p=0.010; salience: PirC, β=0.130, p=0.364; AMY, β=0.179, p=0.271; OFC, β=0.010, p=0.026; VMPFC, β=0.370, p=0.050; one-tailed permutation test). **b.** RSA when pleasantness ratings are discretized with 9 bins. The coefficients for valence and salience are significant in all regions except salience for PirC across n=3 participants. (valence: PirC, β=0.270, p=0.048; AMY, β=0.283, p=0.026; OFC, β=0.470, p=0.000; VMPFC, β=0.430, p=0.000; salience: PirC, β=0.126, p=0.085; AMY, β=0.207, p=0.012; OFC, β=0.297, p=0.000 VMPFC, β=0.298, p=0.000; one-tailed permutation test). Data are presented as mean values and error bars indicate standard error of the mean. Asterisks denote *, p<0.05, **, p<0.01 and ***, p<0.001, respectively.

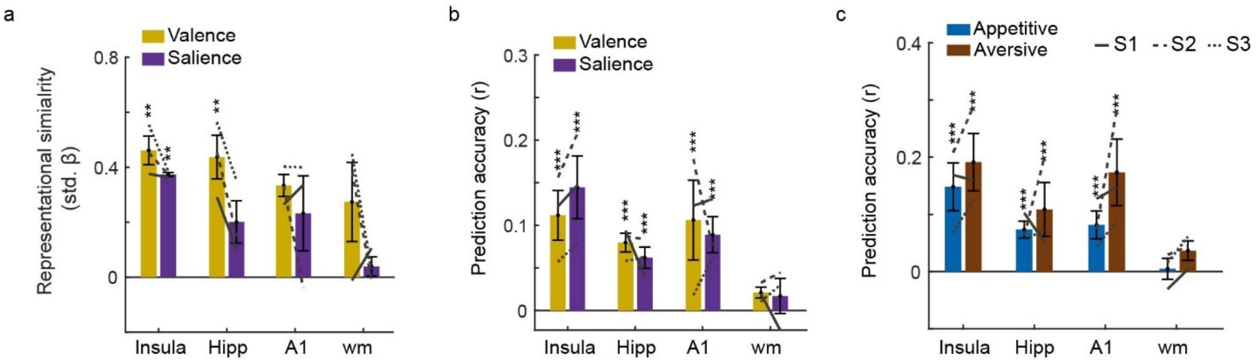


**Supplementary Figure 4.** Effects of valence and salience in other ROIs. **a**. Standardized regression coefficients (beta estimates) for valence and salience from a linear model predicting the neural RSM. The coefficients for valence and salience are significant in Insula, for valence in hippocampus but not elsewhere across n=3 participants. (valence: Insula, β=0.461, p=0.002; Hippocampus, β=0.437, p=0.001; A1, β=0.334, p=0.058; wm, β=0.274, p=0.080; salience: Insula, β=0.374, p=0.003; Hippocampus, β=0.201, p=0.130; A1, β=0.232, p=0.077; wm, β=0.039, p=0.560; one-tailed permutation test). **b.** Average prediction accuracy across n=3 participants (Pearson's correlation between predicted and actual pleasantness in held out test sets) from an SVR model to predict valence and salience. Prediction accuracy is significant in all brain regions except the white matter. (valence: Insula, r=0.112, p<0.001; Hipp, r=0.080, p<0.001; A1, r=0.106, p<0.001; wm, r=0.021, p=0.165; salience: Insula, r=0.145, p<0.001; Hipp, r=0.062, p<0.001; A1, r=0.089, p<0.001; wm, r=0.017, p=0.259; N = 4400; two-tailed t-test).**c**. Average prediction accuracy across n=3 participants (Pearson's correlation between predicted and actual pleasantness in held out test sets) from an SVR model to predict pleasantness of appetitive (or aversive) odors from appetitive (or aversive) odor trials. Prediction accuracy is significant in all brain regions except the white matter. (Appetitive valence: Insula, r=0.148, p<0.001; Hipp, r=0.074, p=0.001; A1, r=0.082, p<0.001; wm, r=0.005, p=0.816; N = 2168; Aversive valence: Insula, r=0.191, p<0.001; Hipp, r=0.109, p<0.001; A1, r=0.173, p<0.001; wm, r=0.037, p=0.084; N = 2177; two-tailed t-test). Data are presented as mean values and error bars indicate standard error of the mean. Asterisks denote *, p<0.05, **, p<0.01 and ***, p<0.001, respectively.

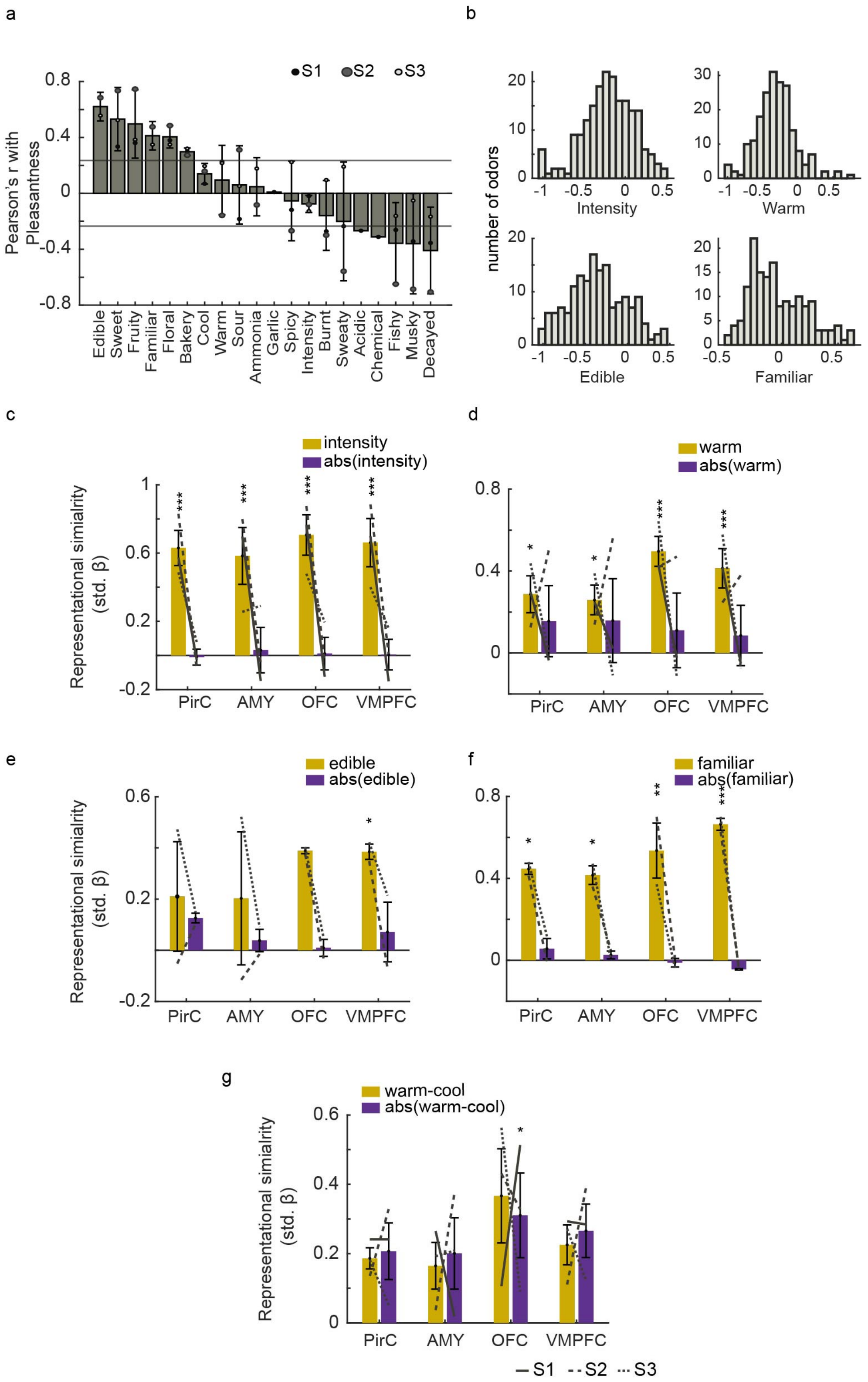


**Supplementary Figure 5**: Representational similarity analysis for perceptual features other than pleasantness. **a**. Pearson's correlation of perceptual ratings with pleasantness. Solid lines indicate significant threshold at $p<0.05$, FWE corrected, two-tailed t-test. **b**. Histograms of ratings of intensity, warm, edible and familiar perceptual features across

odors **c.** Representational similarity analyses performed for intensity and absolute intensity. Standardized regression coefficient is significant for intensity but not absolute intensity in all areas across n=3 participants. (Intensity: PirC, $\beta=0.630$, $p<0.001$; AMY, $\beta=0.585$, $p<0.001$; OFC, $\beta=0.709$, $p<0.001$; VMPFC, $\beta=0.659$, $p<0.001$; absolute Intensity: PirC, $\beta=-0.007$, $p=0.728$; AMY, $\beta=0.032$, $p=0.583$; OFC, $\beta=0.010$, $p=0.762$; VMPFC, $\beta=0.005$, $p=0.718$; one-tailed permutation test). **d**. Representational similarity analyses for warm and absolute warm. Standardized regression coefficient is significant for warm but not absolute warm in all areas across n=3 participants. (warm: PirC, $\beta=0.293$, $p=0.018$; AMY, $\beta=0.256$, $p=0.044$; OFC, $\beta=0.501$, $p<0.001$; VMPFC, $\beta=0.416$, $p=0.001$; absolute warm: PirC, $\beta=0.154$, $p=0.154$; AMY, $\beta=0.157$, $p=0.157$; OFC, $\beta=0.112$, $p=0.112$; VMPFC, $\beta=0.083$, $p=0.083$; one-tailed permutation test). **e.** Representational similarity analyses for edibility and absolute value of edibility. Standardized regression coefficient is not significant for edible or absolute edible in all areas except for edibility in VMPFC and trending in OFC across n=3 participants. (edible: PirC, $\beta=0.214$, p=0. 332; AMY, $\beta=0.207$, p=0. 320; OFC, $\beta=0.389$, $p=0.057$; VMPFC, $\beta=0.383$, p=0. 0.047; absolute edible: PirC, $\beta=0.125$, $p=0.204$; AMY, $\beta=0.035$, $p=0.419$; OFC, $\beta=0.009$, p= 0.484; VMPFC, $\beta=0.078$, $p=0.284$; one-tailed permutation test). **f.** Representational similarity analyses familiarity and absolute value of familiarity. Standardized regression coefficient is significant for familiarity but not absolute familiarity in all areas across n=3 participants. (Familiar: PirC, $\beta=0.442$, p=0. 017; AMY, $\beta=0.410$, p=0. 028; OFC, $\beta=0.533$, $p=0.002$; VMPFC, $\beta=0.661$, p=0. 0.000; absolute familiar: PirC, $\beta=0.052$, $p=0.540$; AMY, $\beta=0.019$, $p=0.628$; OFC, $\beta=-0.011$, p= 0.812; VMPFC, $\beta=-0.051$, $p=0.873$; one-tailed permutation test). Data are presented as mean values and error bars indicate standard error of the mean. Asterisks denote *, $p<0.05$, **, $p<0.01$ and ***, $p<0.001$, respectively. **g**. Representational similarity analyses for warm-cool and absolute values of warm-cool. Standardized regression coefficient is not significant for this composite dimension except for a weak effect for absolute warm-cool in the OFC across n=3 participants. (warm-cool: PirC, $\beta=0.186$, p=0. 293; AMY, $\beta=0.165$, p=0. 378; OFC, $\beta=0.366$, $p=0.057$; VMPFC, $\beta=0.225$, p=0. 0.267; absolute warm-cool: PirC, $\beta=0.207$, $p=0.108$; AMY, $\beta=0.200$, $p=0.128$; OFC, $\beta=0.310$, p= 0.030; VMPFC, $\beta=0.265$, $p=0.060$; one-tailed permutation test).

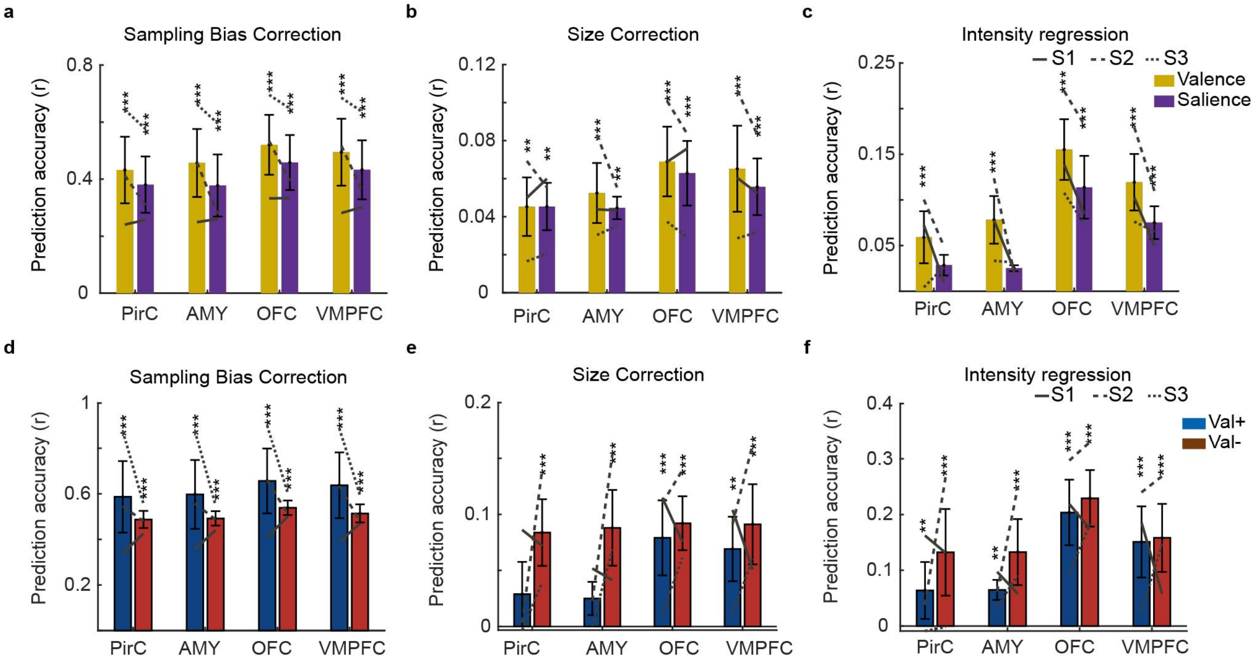


**Supplementary Figure 6. Control decoding analyses to correct for sampling bias, size differences across brain areas, and odor intensity. a.** Correction for differences in sampling biases across different values of pleasantness. We performed a decoding analysis where equal number of appetitive and aversive odors were chosen (N=500). Valence and Salience are significant across all areas. (valence: PirC, r=0.239, p<0.001; AMY, r=0.245, p<0.001; OFC, r=0.295, p<0.001; VMPFC, r=0.276, p<0.001; salience: PirC, r=0.240, p<0.001; AMY, r=0.248, p<0.001; OFC, r=0.307, p<0.001; VMPFC, r=0.270, p<0.001; N = 1000; two-tailed t-test). **b**. Correction for differences in voxels across sizes. Same number of voxels (=100) were chosen across all ROIs. (valence: PirC, r=0.045, p=0.003; AMY, r=0.053, p=0.005; OFC, r=0.069, p<0.001; VMPFC, r=0.065, p<0.001; salience: PirC, r=0.045, p=0.003; AMY, r=0.045, p=0.003; OFC, r=0.063, p<0.001; VMPFC, r=0.056, p<0.001; N = 4400; two-tailed t-test). **c**. We regressed out intensity ratings from the valence and salience prior to decoding. (valence: PirC, r=0.059, p<0.001; AMY, r=0.078, p<0.001; OFC, r=0.155, p<0.001; VMPFC, r=0.119, p<0.001; salience: PirC, r=0.028, p=0.059; AMY, r=0.025, p=0.096; OFC, r=0.114, p<0.001; VMPFC, r=0.075, p<0.001; N = 4400; two-tailed t-test). **d.** Appetitive and aversive pleasantness. Sampling bias correction to correct for imbalance across number of odors belonging to appetitive vs. aversive odors. Equal number of odors (N=500) were chosen for both sets. (Appetitive valence: PirC, r=0.231, p<0.001; AMY, r=0.231, p<0.001; OFC, r=0.304, p<0.001; VMPFC, r=0.292, p<0.001; N = 500; Aversive valence: PirC, r=0.291, p<0.001; AMY, r=0.292, p<0.001; OFC, r=0.326, p<0.001; VMPFC, r=0.298, p<0.001; N = 500; two-tailed t-test). **e.** Correction for differences in voxels across sizes. Same number of voxels (=100) were chosen across all ROIs. (Appetitive valence: PirC, r=0.029, p=0.170; AMY, r=0.025, p=0.237; OFC, r=0.079, p<0.001; VMPFC, r=0.069, p=0.001; N = 2168; Aversive valence: PirC, r=0.084, p<0.001; AMY, r=0.088, p<0.001; OFC, r=0.092, p<0.001; VMPFC, r=0.091, p<0.001; N = 2177; two-tailed t-test) **f**. We regressed out intensity ratings from the valence and salience prior to decoding (Appetitive valence: PirC, r=0.064, p=0.003; AMY, r=0.065, p=0.003; OFC, r=0.204, p<0.001; VMPFC, r=0.151, p<0.001; N = 2168; Aversive valence: PirC, r=0.132, p<0.001; AMY, r=0.133, p<0.001; OFC, r=0.229, p<0.001; VMPFC, r=0.158, p<0.001; N = 2177; two-tailed t-test). Data are presented as mean values and error bars indicate standard error of the mean. Asterisks denote *, p<0.05, **, p<0.01 and ***, p<0.001, respectively.

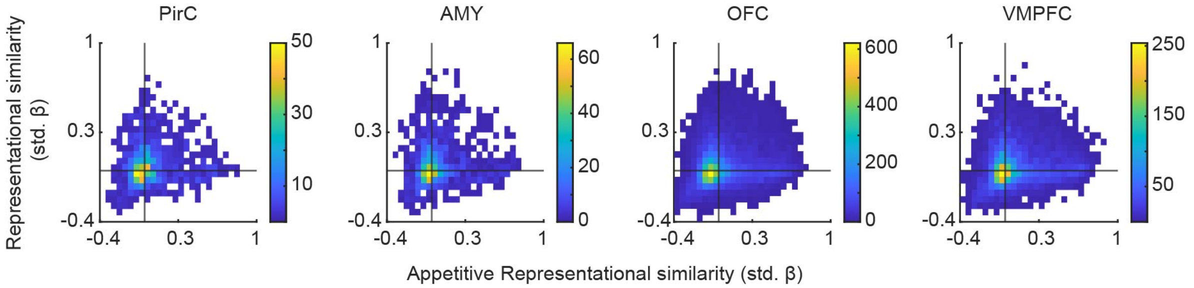


**Supplementary Figure 7:** Distribution of voxel-wise RSA coefficients for the pleasantness of appetitive and aversive odors. Hot colors indicate higher density of voxels. The highest density is close to the central bins (0,0).

**SUPPLEMENTARY TABLES**

| CID | Odor name | S1 | S2 | S3 |
|---|---|---|---|---|
| 126 | 4-Hydroxybenzaldehyde | -0.706 | N/A | N/A |
| 177 | acetaldehyde | -0.249 | N/A | N/A |
| 196 | Adipic acid | 0.192 | N/A | N/A |
| 239 | beta-Alanine | 0.014 | N/A | N/A |
| 240 | benzaldehyde | 0.79 | -0.044 | -0.186 |
| 261 | butyraldehyde | -0.439 | -0.525 | -0.158 |
| 263 | butanol | N/A | -0.413 | -0.347 |
| 325 | 4-Isopropylbenzyl alcohol | 0.334 | -0.204 | -0.175 |
| 326 | cuminaldehyde | -0.715 | -0.26 | -0.333 |
| 356 | Octane | 0.186 | -0.167 | 0.483 |
| 379 | Octanoic acid | -0.127 | N/A | N/A |
| 460 | Guaiacol | -0.02 | -0.138 | -0.383 |
| 650 | 2,3-butanedione | -0.042 | 0.625 | 0.033 |
| 660 | Dihydrocoumarin | -0.422 | -0.131 | -0.008 |
| 957 | octanol | 0.482 | -0.142 | 0.031 |
| 1001 | Phenethylamine | 0.145 | -0.179 | -0.289 |
| 1032 | propionic acid | N/A | -0.327 | -0.558 |
| 1049 | pyridine | N/A | -0.265 | 0.328 |
| 1068 | methyl sulfide | -0.919 | -0.875 | -0.675 |
| 1110 | Succinic acid | -0.447 | N/A | N/A |
| 1136 | 4-Methyl-5-thiazoleethanol | 0.304 | -0.313 | -0.456 |
| 2214 | Acetovanillone | 0.347 | N/A | N/A |
| 2345 | Benzyl benzoate | 0.132 | -0.123 | -0.022 |
| 2346 | Benzyl isothiocyanate | 0.105 | N/A | N/A |
| 2758 | 1,8-cineole | 0.951 | -0.367 | -0.036 |
| 2879 | p-cresol | -0.24 | -0.519 | -0.931 |
| 2969 | decanoic acid | 0.088 | N/A | N/A |
| 3893 | Lauric acid (replicate) | -0.094 | N/A | N/A |
| 4133 | methyl salicylate | 0.165 | -0.054 | 0.256 |
| 5541 | Triacetin | 0.217 | N/A | N/A |
| 5610 | Tyramine | 0.045 | N/A | N/A |
| 5779 | D-(-)-Ribose | 0.23 | N/A | N/A |
| 5780 | D-Sorbitol | 0.368 | N/A | N/A |
| 5960 | Aspartic Acid | 0.001 | N/A | N/A |
| 6050 | Tributyrin | 0.127 | N/A | N/A |
| 6054 | 2-phenylethanol | N/A | -0.465 | 0.358 |
| 6106 | L-Leucine | 0.056 | N/A | N/A |
| 6184 | Hexanal | 0.424 | -0.221 | -0.119 |

| | | | | |
|---|---|---|---|---|
| 6448 | Bornyl acetate | N/A | -0.227 | 0.072 |
| 6501 | Ethyl 3-methyl-3-phenylglycidate | N/A | -0.381 | 0.092 |
| 6549 | Linalool | 0.424 | -0.173 | 1 |
| 6584 | methyl acetate | -0.301 | -0.265 | -0.694 |
| 6590 | Isobutyric acid | 0.239 | -0.456 | 0.261 |
| 6658 | Methyl methacrylate | N/A | -0.433 | -0.489 |
| 6943 | 2-Isopropylphenol | -0.24 | -0.238 | 0.008 |
| 6997 | 2-ethylphenol | -0.355 | -0.423 | -0.153 |
| 6998 | salicylaldehyde | -0.193 | 0.304 | -0.069 |
| 7059 | 6-methyl quinoline | -0.006 | N/A | N/A |
| 7095 | Biphenyl | -0.076 | N/A | N/A |
| 7122 | Methyl beta-naphthyl ketone | 0.114 | N/A | N/A |
| 7136 | Eugenyl Acetate | 0.519 | -0.013 | -0.136 |
| 7144 | 2-Methoxy-4-methylphenol (replicate) | -0.456 | -0.046 | -0.042 |
| 7151 | Methyl nicotinate | -0.055 | -0.398 | -0.397 |
| 7165 | Ethyl Benzoate | 0.259 | -0.038 | 0.247 |
| 7335 | carvyl acetate | N/A | -0.55 | -0.378 |
| 7361 | Furfuryl alcohol | 0.269 | N/A | N/A |
| 7410 | acetophenone | 0.519 | -0.206 | -0.272 |
| 7463 | para-cymene | -0.378 | -0.069 | -0.219 |
| 7500 | ethylbenzene | -0.928 | -0.669 | -0.031 |
| 7519 | anisole | -0.62 | -0.425 | -0.128 |
| 7593 | 1,3-Diphenyl-2-propanone | -0.564 | -0.133 | 0.256 |
| 7635 | 2-ethylhexyl acetate | 0.196 | -0.01 | 0.189 |
| 7654 | Phenethyl acetate | 0.882 | -0.129 | 0.172 |
| 7720 | 2-ethyl-1-hexanol | N/A | -0.49 | 0.2 |
| 7731 | 4-Methylanisole | 0.111 | -0.523 | 0.253 |
| 7749 | ethyl propionate | 0.476 | -0.388 | 0.153 |
| 7761 | Diethyl malonate | 0.606 | -0.258 | 0.292 |
| 7762 | ethyl butyrate | -0.309 | -0.54 | 0.936 |
| 7795 | Isoamyl butyrate | -0.65 | 0.175 | 0.217 |
| 7799 | Ethyl octanoate | 0.606 | -0.156 | 0.158 |
| 7803 | Propyl propionate | -0.131 | -0.423 | 0.019 |
| 7824 | methyl caproate | N/A | -0.367 | 1 |
| 7937 | 2,6-Lutidine | N/A | -0.517 | -0.497 |
| 7967 | Cyclohexanone | 0.101 | -0.038 | -0.056 |
| 7983 | butyl butyrate | 0.168 | -0.292 | 0.547 |
| 7991 | valeric acid | N/A | -0.467 | -0.189 |
| 8077 | Diethyl disulfide | -0.114 | -0.944 | -1 |
| 8082 | Piperidine | -0.353 | -0.246 | -0.078 |
| 8093 | 2-octanone | 0.206 | -0.058 | 0.092 |

| | | | | |
|---|---|---|---|---|
| 8103 | hexanol | 0.64 | -0.196 | -0.236 |
| 8118 | propyl sulfide | N/A | -0.254 | -0.594 |
| 8129 | heptanol | 0.905 | -0.431 | N/A |
| 8174 | decanol | 0.286 | N/A | N/A |
| 8184 | 1-undecanol | -0.534 | -0.054 | 0.108 |
| 8186 | undecanal | 0.482 | -0.102 | -0.25 |
| 8193 | Lauryl alcohol | 0.148 | N/A | N/A |
| 8375 | 2-Hydroxyacetophenone | 0.157 | -0.248 | -0.061 |
| 8456 | Butylated hydroxyanisole | -0.378 | N/A | N/A |
| 8658 | o-Anisaldehyde | 0.105 | N/A | N/A |
| 8697 | 2-Ethylhexanoic acid | 0.45 | N/A | N/A |
| 8712 | o-Toluenethiol | 0.424 | -0.456 | -0.811 |
| 8723 | 2-Methyl-1-butanol (replicate) | -0.606 | -0.563 | 0.172 |
| 8785 | benzyl acetate | -0.353 | -0.217 | 0.547 |
| 8797 | p-Tolyl acetate | 0.141 | -0.25 | -0.158 |
| 8857 | ethyl acetate | N/A | -0.248 | -0.075 |
| 8892 | caproic acid | N/A | -0.25 | -0.372 |
| 8908 | hexyl acetate | 0.23 | 0.083 | 0.183 |
| 8914 | nonanol | -0.832 | -0.069 | -0.161 |
| 8918 | Nonyl acetate | N/A | -0.05 | -0.036 |
| 9025 | 1,3-Dimethoxybenzene | 0.304 | -0.063 | 0.214 |
| 9589 | 3-Acetylpyridine | -0.056 | -0.423 | -0.122 |
| 9609 | Diethyl sulfide | -0.348 | -0.85 | -0.997 |
| 9862 | 6-Methyl-5-hepten-2-one | 0.904 | 0.14 | 0.081 |
| 10364 | Carvacrol | 0.071 | -0.154 | 0.083 |
| 10400 | Cycloheptanone | 0.718 | -0.233 | 0.075 |
| 10430 | isovaleric acid | N/A | -0.521 | -0.072 |
| 10448 | 3-(methylthio)-1-propanol | 0.027 | -0.381 | -0.472 |
| 10882 | Ethyl Valerate | 0.738 | -0.208 | 0.856 |
| 10890 | Amyl butyrate | -0.176 | -0.471 | 0.278 |
| 10895 | Isobutyl propionate | 0.217 | -0.379 | 0.544 |
| 10925 | 3-Methyl-1-butanethiol | -0.37 | -0.552 | -0.717 |
| 11086 | 2-Aminoacetophenone | 0.127 | -0.125 | 0.158 |
| 11428 | 3-pentanol | -0.695 | -0.679 | -0.444 |
| 11525 | 4-Methylcyclohexanone | 0.01 | -0.129 | -0.147 |
| 11527 | 3-Octanol | -0.442 | -0.133 | -0.206 |
| 11529 | Butyl Propionate | 0.77 | -0.167 | 0.228 |
| 11569 | 3-methoxy benzaldehyde | N/A | -0.392 | -0.208 |
| 11583 | 2-hexanone | 0.459 | -0.067 | 0.461 |
| 11614 | Butyl formate | 0.23 | N/A | N/A |
| 11902 | methyl 2-furoate | -0.005 | -0.113 | -0.269 |

| | | | | |
|---|---|---|---|---|
| 11980 | Ethyl 2-furoate | 0.413 | N/A | N/A |
| 12180 | Methyl butyrate | N/A | -0.546 | -0.025 |
| 12297 | 2-hexanol | 0.105 | -0.238 | -0.097 |
| 12327 | Ethyl undecanoate | -0.085 | N/A | N/A |
| 12587 | 4-Methylvaleric acid | -0.188 | -0.452 | 0.681 |
| 12741 | 2-decanone | 0.168 | -0.129 | -0.042 |
| 12810 | delta-Decalactone | -0.068 | -0.392 | -0.072 |
| 12813 | gamma-decalactone | 0.333 | -0.056 | -0.036 |
| 14257 | undecane | -0.711 | N/A | N/A |
| 14286 | 2-acetyl pyridine | 0.81 | -0.31 | -0.022 |
| 14296 | 2,3,5,6-Tetramethylpyrazine | -0.978 | -0.496 | -0.167 |
| 14491 | 1,6-Hexanedithiol | 0.191 | -0.44 | -0.042 |
| 14514 | 2-Acetyl-5-methylfuran | N/A | -0.265 | -0.167 |
| 14525 | l-fenchone | 0.588 | N/A | N/A |
| 15037 | 1-Furfurylpyrrole | -0.594 | -0.313 | -0.522 |
| 15380 | Bis(methylthio)methane | N/A | -0.498 | -0.872 |
| 15510 | Cyclopentanethiol | N/A | -0.588 | -1 |
| 16741 | 2-Phenylethyl isothiocyanate | N/A | -0.192 | 0.056 |
| 17525 | hexyl butyrate | N/A | -0.25 | -0.108 |
| 17617 | Allyl cyclohexanepropionate | 0.096 | -0.392 | 0.156 |
| 18635 | 3-(Methylthio)propionaldehyde | 0.128 | -0.275 | -0.719 |
| 18827 | 1-Octen-3-ol | -0.609 | -0.24 | -0.253 |
| 21057 | 1-Hepten-3-ol | -0.94 | -0.438 | -0.319 |
| 21648 | 4-(4-Hydroxyphenyl)-2-butanone | 0.588 | N/A | N/A |
| 22873 | Hexyl hexanoate | -0.511 | -0.125 | -0.133 |
| 23235 | Hexyl benzoate | N/A | -0.531 | -0.328 |
| 23642 | 2-Methoxythiophenol | 0.062 | -0.196 | -0.386 |
| 24834 | 4-Ethoxybenzaldehyde | 0.062 | N/A | -0.006 |
| 26331 | 2-ethyl pyrazine | 0.134 | -0.563 | -0.031 |
| 27458 | 2,3-Diethylpyrazine | 0.321 | -0.252 | -0.258 |
| 31209 | alpha-Amylcinnamaldehyde | 0.295 | -0.246 | -0.153 |
| 31234 | 3-Phenyl-1-propanol | 0.364 | -0.435 | 0.197 |
| 31244 | p-Anisaldehyde | 0.519 | -0.117 | -0.322 |
| 31246 | 4-Heptanone | 0.787 | -0.244 | 0.636 |
| 31249 | diethyl succinate | 1 | -0.142 | 0.817 |
| 31260 | isoamyl alcohol | N/A | -0.325 | -0.369 |
| 31265 | ethyl hexanoate | 0.58 | -0.242 | 1 |
| 31272 | butyl acetate | 0.358 | -0.319 | 0.331 |
| 32594 | 2-isobutyl-3-methoxypyrazine | -0.176 | -0.254 | -0.244 |
| 36822 | 5,6,7,8-Tetrahydroquinoxaline | 0.252 | -0.223 | 0.131 |
| 61048 | Isobutyl benzoate | N/A | -0.4 | -0.139 |

| | | | | |
|---|---|---|---|---|
| 61052 | 3-Phenylpropyl propionate | N/A | -0.256 | -0.111 |
| 61138 | 4-Pentenoic acid | -0.03 | -0.317 | -0.161 |
| 61199 | 5-Ethyl-3-hydroxy-4-methyl-2(5H)-furanone | 0.062 | 0.246 | 0.006 |
| 61204 | delta-Undecalactone | N/A | N/A | -0.044 |
| 61527 | 3-Acetyl-2,5-dimethylfuran | -0.404 | -0.36 | -0.392 |
| 61653 | 2,4,5-Trimethylthiazole | -0.468 | -0.431 | -0.267 |
| 61670 | 5-Methylquinoxaline | 0.275 | 0.004 | -0.033 |
| 61918 | 2-Acetyl-3-ethylpyrazine | -0.459 | -0.533 | -0.189 |
| 62375 | Benzaldehyde dimethyl acetal | 0.295 | -0.452 | -0.286 |
| 62378 | Dihydrojasmone | 0.437 | -0.033 | -0.242 |
| 62444 | Methyl thiobutyrate | -1 | -1 | -0.983 |
| 62835 | 4,5-Dimethyl-3-hydroxy-2,5-dihydrofuran-2-one | 0.299 | 0.2 | 0.092 |
| 62902 | Ethyl 2-methylpentanoate | 0.574 | -0.077 | 1 |
| 78925 | 2-(Methylthio)ethanol | N/A | -0.494 | -0.744 |
| 82227 | alpha-pinene | 0.157 | -0.181 | -0.164 |
| 89440 | 6-Acetyl-1,1,2,4,4,7-Hexamethyltetralin | 0.281 | N/A | N/A |
| 170833 | (-)-Isopulegol | 0.64 | -0.319 | -0.161 |
| 235414 | Omega-Pentadecalactone | -0.314 | -0.069 | -0.094 |
| 246728 | 3-octanone | N/A | -0.027 | -0.189 |
| 439570 | (-)-Carvone | 0.517 | -0.26 | 0.364 |
| 440967 | (-)-beta-Pinene | 0.563 | -0.225 | 0.386 |
| 444539 | trans-Cinnamic acid | -0.145 | N/A | N/A |
| 520191 | 3-Acetyl-2,5-dimethylthiophene | -0.465 | -0.317 | 0.208 |
| 556940 | Ethyl 3-(furfurlthio) propionate | N/A | -0.125 | -0.117 |
| 637563 | trans-Anethole | 0.066 | -0.123 | -0.097 |
| 637566 | geraniol | N/A | -0.223 | -0.161 |
| 637796 | Isosafrole | 0.252 | -0.244 | -0.294 |
| 638014 | beta-Ionone | 0.796 | -0.188 | -0.033 |
| 643820 | Nerol | N/A | -0.25 | 0.433 |
| 3578033 | 3-(5-Methyl-2-furyl)butanal | 0.247 | -0.419 | -1 |
| 5315892 | cinnamyl alcohol | 0.282 | N/A | N/A |
| 5365027 | cis-2-Nonen-1-ol | N/A | -0.242 | -0.819 |
| 5366244 | Phytol | 0.465 | -0.277 | -0.333 |
| 6999977 | Butyl (S)-(-)-lactate | -0.435 | -0.108 | -0.006 |

**Supplementary Table 1. Average odor pleasantness ratings per participant.**

| | | Valence | Salience | Appetitive | Aversive | Cross decoding |
|---|---|---|---|---|---|---|
| S1 | PirC | 0.000 | 0.000 | 0.000 | 0.000 | 0.010 |
| | AMY | 0.000 | 0.000 | 0.000 | 0.000 | 0.005 |
| | OFC | 0.000 | 0.000 | 0.000 | 0.000 | 0.000 |
| | VMPFC | 0.000 | 0.000 | 0.000 | 0.001 | 0.019 |
| S2 | PirC | 0.000 | 0.000 | 0.635 | 0.000 | 0.027 |
| | AMY | 0.000 | 0.000 | 0.005 | 0.000 | 0.873 |
| | OFC | 0.000 | 0.000 | 0.000 | 0.000 | 0.038 |
| | VMPFC | 0.000 | 0.000 | 0.000 | 0.000 | 0.170 |
| S3 | PirC | 0.007 | 0.457 | 0.248 | 0.006 | 0.672 |
| | AMY | 0.000 | 0.000 | 0.115 | 0.000 | 0.563 |
| | OFC | 0.000 | 0.000 | 0.010 | 0.000 | 0.148 |
| | VMPFC | 0.001 | 0.000 | 0.339 | 0.000 | 0.305 |

**Supplementary Table 2:** P-values from participant-wise statistical tests of decoding analyses for valence and salience in Fig 3b, appetitive and aversive value coding in Fig 4b and cross-decoding analyses in Fig 5b.

| | | Valence | Salience | Appetitive | Aversive | Cross decoding |
|---|---|---|---|---|---|---|
| S1 | PirC | 0.065 | 0.104 | 0.168 | 0.146 | -0.054 |
| | AMY | 0.083 | 0.089 | 0.083 | 0.095 | -0.059 |
| | OFC | 0.101 | 0.166 | 0.213 | 0.139 | -0.127 |
| | VMPFC | 0.095 | 0.097 | 0.217 | 0.069 | -0.049 |
| S2 | PirC | 0.106 | 0.102 | 0.010 | 0.267 | -0.047 |
| | AMY | 0.153 | 0.087 | 0.061 | 0.262 | -0.003 |
| | OFC | 0.245 | 0.223 | 0.303 | 0.356 | -0.045 |
| | VMPFC | 0.190 | 0.168 | 0.228 | 0.258 | -0.030 |
| S3 | PirC | 0.041 | 0.011 | -0.025 | 0.061 | -0.009 |
| | AMY | 0.072 | 0.066 | 0.034 | 0.165 | -0.013 |
| | OFC | 0.090 | 0.082 | 0.055 | 0.140 | -0.031 |
| | VMPFC | 0.053 | 0.092 | 0.021 | 0.159 | -0.022 |

**Supplementary Table 3:** Effect sizes (Pearson's r) of participant-wise statistical tests of decoding analyses for valence and salience in Fig 3b, appetitive and aversive value coding in Fig 4b and cross-decoding analyses in Fig 5b.
