# Supplementary material for: Separable and integrated pleasantness coding for appetitive and aversive odors across olfactory and ventral prefrontal cortices
Source: Nat Commun. 2026 May 22;17:6732. doi: 10.1038/s41467-026-73001-7 (PMC13385377; doi:10.1038/s41467-026-73001-7)
Supplement: Supplementary file 2 — Reporting Summary [file 41467_2026_73001_MOESM2_ESM.pdf]

nature portfolio

Corresponding author(s): Thorsten Kahnt

Last updated by author(s): Mar 19, 2026

Reporting Summary

Nature Portfolio wishes to improve the reproducibility of the work that we publish. This form provides structure for consistency and transparency in reporting. For further information on Nature Portfolio policies, see our Editorial Policies and the Editorial Policy Checklist.

Statistics

For all statistical analyses, confirm that the following items are present in the figure legend, table legend, main text, or Methods section.

n/a	Confirmed	
☐	☒	The exact sample size (*n*) for each experimental group/condition, given as a discrete number and unit of measurement
☐	☒	A statement on whether measurements were taken from distinct samples or whether the same sample was measured repeatedly
☐	☒	The statistical test(s) used AND whether they are one- or two-sided<br>*Only common tests should be described solely by name; describe more complex techniques in the Methods section.*
☐	☒	A description of all covariates tested
☐	☒	A description of any assumptions or corrections, such as tests of normality and adjustment for multiple comparisons
☐	☒	A full description of the statistical parameters including central tendency (e.g. means) or other basic estimates (e.g. regression coefficient) AND variation (e.g. standard deviation) or associated estimates of uncertainty (e.g. confidence intervals)
☐	☒	For null hypothesis testing, the test statistic (e.g. *F*, *t*, *r*) with confidence intervals, effect sizes, degrees of freedom and *P* value noted<br>*Give P values as exact values whenever suitable.*
☒	☐	For Bayesian analysis, information on the choice of priors and Markov chain Monte Carlo settings
☒	☐	For hierarchical and complex designs, identification of the appropriate level for tests and full reporting of outcomes
☐	☒	Estimates of effect sizes (e.g. Cohen's *d*, Pearson's *r*), indicating how they were calculated

Our web collection on statistics for biologists contains articles on many of the points above.

Software and code

Policy information about availability of computer code

Data collection	Custom code, COGENT 2000, MATLAB R2016b
Data analysis	Custom code, SPM 12, MATLAB R2020b, MATLAB R2023b, LibSVM, GLMSingle Package, Representational Similarity Analysis, Support Vector Machine, Breathmetrics.<br>Custom code is available at https://github.com/viveksgr/ARC and archived on Zenodo https://doi.org/10.5281/zenodo.19119376

For manuscripts utilizing custom algorithms or software that are central to the research but not yet described in published literature, software must be made available to editors and reviewers. We strongly encourage code deposition in a community repository (e.g. GitHub). See the Nature Portfolio guidelines for submitting code & software for further information.

Data

Policy information about availability of data

All manuscripts must include a data availability statement. This statement should provide the following information, where applicable:

- Accession codes, unique identifiers, or web links for publicly available datasets
- A description of any restrictions on data availability
- For clinical datasets or third party data, please ensure that the statement adheres to our policy

Dataset to reproduce all major findings of the study is available without restriction at https://github.com/viveksgr/ARC and archived on Zenodo https://doi.org/10.5281/zenodo.19119376. Analyses presented in this manuscript are based on previously published dataset https://doi.org/10.5281/zenodo.7636722.

The access request to the raw dataset can be submitted at Zenodo and is subject to a data-use agreement that restricts use to research purposes, prohibits re-identification and redistribution, and requires citation of the associated Zenodo DOI and publication. The timeframe for response to requests is approximately 10 business days.

Research involving human participants, their data, or biological material

Policy information about studies with human participants or human data. See also policy information about sex, gender (identity/presentation), and sexual orientation and race, ethnicity and racism.

Reporting on sex and gender	The study involved 3 human subjects (2 Female, 1 Male). Participants were asked to self-identify their biological sex with an option to not report it. The demographic survey was based on NIH guidelines and approved by IRB. We did not collect gender information since we did not consider that gender was likely to influence our main findings. We did not carry out sex/gender-based analysis because sex/gender was unlikely to influence our main findings.
Reporting on race, ethnicity, or other socially relevant groupings	Subjects were asked to report their race and ethnicity (or prefer not to answer). The demographic survey was based on NIH guidelines and approved by IRB. We did not consider race or ethnicity as a proxy for socioeconomic status. We did not perform any analyses that compared influence of race or ethnicity on our findings since the primary focus of this experiment was to study how odor pleasantness underlies the synthesis of odor valence and salience in the brain which was likely to be unaffected by race or ethnicity.
Population characteristics	Sample consisted of three healthy human subjects (2 females ages 23-24, 1 male age 24). Subjects were right-handed, native English speakers with normal or corrected to normal vision. Subjects had no history of prior psychiatric or neurological disorders, no significant medical disorders, no smell and taste dysfunction or a history of sinusitis or allergic rhinitis and were not using medications that could affect alertness.
Recruitment	In the original study, subjects were recruited through the Kahnt Lab Research Registry. Informed consent was obtained from each subject. The subjects and investigators were not acquainted prior to the study, and investigators report no self-selection biases. Subjects received monetary compensation amounting to \$40 per hour for sessions involving fMRI (18 hours), \$20 per hour for behavioral sessions outside the scanner (12-16 hours) and a study completion bonus of \$300.
Ethics oversight	The study protocol was approved by Northwestern University's Institutional Review Board.

Note that full information on the approval of the study protocol must also be provided in the manuscript.

Field-specific reporting

Please select the one below that is the best fit for your research. If you are not sure, read the appropriate sections before making your selection.

☒ Life sciences ☐ Behavioural & social sciences ☐ Ecological, evolutionary & environmental sciences

For a reference copy of the document with all sections, see nature.com/documents/nr-reporting-summary-flat.pdf

Life sciences study design

All studies must disclose on these points even when the disclosure is negative.

Sample size	160 odor stimuli (conditions, 27-30 repetitions each) were tested in 3 independent subjects. No sample size estimation was done. We chose a precision approach involving many stimuli and repetitions in a small number of subjects, and statistics were computed within subjects.
Data exclusions	No data was excluded from the previously published dataset. Original dataset removed one additional subject prior to analysis due to a psychiatric disorder that was disclosed after data collection.
Replication	Each of the 160 odor stimuli were repeated 27-30 times in each of the three subjects.
Randomization	In this study with a within-subject design, the order of the stimuli was randomized across experimental sessions.
Blinding	Single-blind. Subjects were blinded with respect to the order of the conditions.

Reporting for specific materials, systems and methods

We require information from authors about some types of materials, experimental systems and methods used in many studies. Here, indicate whether each material, system or method listed is relevant to your study. If you are not sure if a list item applies to your research, read the appropriate section before selecting a response.

Materials & experimental systems

n/a	Involved in the study
☒	☐ Antibodies
☒	☐ Eukaryotic cell lines
☒	☐ Palaeontology and archaeology
☒	☐ Animals and other organisms
☒	☐ Clinical data
☒	☐ Dual use research of concern
☒	☐ Plants

Methods

n/a	Involved in the study
☒	☐ ChIP-seq
☒	☐ Flow cytometry
☐	☒ MRI-based neuroimaging

Plants

Seed stocks

N/A

Novel plant genotypes

N/A

Authentication

N/A

Magnetic resonance imaging

Experimental design

Design type

Task-based event-related design.

Design specifications

Each subject was presented with 160 odor stimuli, each repeated 27-30 times. There were 12 fMRI sessions per subject, each up to 2 hours in duration and consisted of 4 runs. Each run consisted of 10 odor stimuli presented across 90-100 trials. Each odor stimulus was presented in 3 sessions resulting in 27-30 trials per stimulus per subject.

Behavioral performance measures

In each trial, subjects responded if they could smell the odor stimulus. If they could, they rated the odor on one perceptual descriptor. Their rating, button press and reaction times were recorded. Consistency of perceptual ratings over repeated presentations of the odor stimuli was quantified using Pearson's correlation.

Acquisition

Imaging type(s)

Functional and Structural

Field strength

3T

Sequence & imaging parameters

Gradient echo T2* weighted echoplanar images were acquired during the functional scan. For subject 1: repetition time (TR) = 1.4 seconds, echo time (TE) = 22ms, matrix size = 104×96 voxels, flip angle = 80°, in-plane resolution = 2×2mm, slice thickness = 2mm, multiband factor (MB) = 2, 42 slices. To further optimize the spatial coverage in subjects 2 and 3, small adjustment to the scanning sequence were made (TE = 24ms, flip angle = 70°, matrix size = 122×102 voxels, in-plane resolution = 1.7×1.7mm, slice thickness = 2mm, MB = 3, 38 slices). High-resolution T1 weighed anatomical images (1mm3 isotropic) were acquired for anatomical localization with the following parameters: TR = 2170 ms; TE = 1.69 ms; flip angle, 7˚, 1 mm isotropic voxels, no gap, number of slices = 256; field of view= 176 mm x 256 mm x 256 mm).

Area of acquisition

The imaging sequence was optimized for signal recovery in olfactory areas. Functional images were acquired at an acquisition angle= 30° rostral to the inter-commissural line, slices per image = 42 in subject 1 and slices per image = 38 in subject 2 and 3.

Diffusion MRI ☐ Used ☒ Not used

Preprocessing

Preprocessing software

Default functions in SPM12 were used to realign the functional images and coregister them to the anatomical image. Functional images were smoothed using a 2mm3 FWHM gaussian kernel. The anatomical image was segmented to extract gray matter voxels. GLMsingle package was used to extract single trial responses for each voxel.

Normalization

Data was not normalized. All analyses were performed in the native space of the subject since the study does not involve estimation of any population level brain maps.

Normalization template	Data was not normalized. However, Montreal Neurological Institute (MNI) atlas was used to draw ROIs that were inverse-normalized to subjects' native space.
Noise and artifact removal	Translation and rotation parameters estimated during the realignment procedure were used as nuisance regressors to account for motion-related effects. Additional nuisance regressors were appended to account for the difference and variance in interleaved sets of slices within volumes. In order to account for sniff-related effects, sniff traces obtained from breathing measurements were appended as nuisance regressors.
Volume censoring	Volume censoring was not performed for these analyses since the data was based on subjects wearing custom headcases which allowed only minimal head motion.

Statistical modeling & inference

Model type and settings	Representational Similarity Analysis (RSA) and decoding analyses based on support vector machine (SVM)
Effect(s) tested	Standardized regression coefficient of neural similarity in an ROI regressed against similarity in aspects of pleasantness; Pearson's correlation of pleasantness-related predictions by SVM with actual values on a trial .

Specify type of analysis: ☐ Whole brain ☒ ROI-based ☐ Both

Anatomical location(s)	We focused on a set of olfactory and ventral prefrontal regions of interest (ROI), namely the piriform cortex (PirC), amygdala (AMY), orbitofrontal cortex (OFC), and ventromedial prefrontal cortex (VMPFC).
Statistic type for inference (See Eklund et al. 2016)	ROI-wise and searchlight based analyses
Correction	Brain maps were corrected for multiple comparisons using FDR

Models & analysis

n/a	Involved in the study
☒	☐ Functional and/or effective connectivity
☒	☐ Graph analysis
☐	☒ Multivariate modeling or predictive analysis

Multivariate modeling and predictive analysis	Searchlight and ROI based approach was used to relate the similarity of neural data with similarity in aspects of pleasantness (e.g. appetitive or aversive domains).
