# Supplementary material for: Separable and integrated pleasantness coding for appetitive and aversive odors across olfactory and ventral prefrontal cortices
Source: Nat Commun. 2026 May 22;17:6732. doi: 10.1038/s41467-026-73001-7 (PMC13385377; doi:10.1038/s41467-026-73001-7)
Supplement: Supplementary file 3 — Transparent Peer Review file [file 41467_2026_73001_MOESM3_ESM.pdf]

nature portfolio

Peer Review File

Separable and integrated pleasantness coding for appetitive and aversive odors across olfactory and ventral prefrontal cortices

Corresponding Author: Dr Thorsten Kahnt

This file contains all reviewer reports in order by version, followed by all author rebuttals in order by version.

Version 0:

Reviewer comments:

Reviewer #1

(Remarks to the Author)
The study by Sagar et al. addresses a very important question in olfaction regarding the major axis of perception. In his previous paper, Sagar et al, [Nature Neuroscience 2023] showed that the encoded perceptual spaces increase in dimensionality and subjectivity from the PirC to the OFC. In the current paper the same data has been used to test how pleasantness of aversive and appetitive odors is represented in olfactory regions.
The authors used a similar modeling approach from their previous publication [Kahnt et al, 2014], however, I have a few questions regarding the use of this model in olfaction. First, subjects rated pleasantness, why using the model and not the real pleasantness rating average? Second, sniffing is modulated by subjective pleasantness of an odor, and a mere sniff induce activation if olfactory areas. Although the sniff flow was treated as nuisance regressor, I am not sure this correction method is effective. I like to see figures with sniff flow vs. pleasantness ratings for each subject. Third, the correlations values are low, for a control, please repeat the analysis with intensity / familiarity / cool-warm (assuming similar gaussian distribution as in Extended Data Figure 1). Finally, regarding the selected ROIs, other relevant regions that should be addressed are the insula, hippocampus, and medial prefrontal cortex, as well as auditory cortex (A1) and white matter (to rule out statistical bias in the analysis). A different approach will be searchlight analysis (as in Kahnt et al 2014).
typo: "In P0 voxels, prediction accuracy was significant for both <appetitive> and appetitive odors in the OFC"
"<and> but not in PirC and AMY"

Reviewer #2

(Remarks to the Author)
The manuscript "Separable and integrated pleasantness coding for appetitive and aversive odors across olfactory and ventral prefrontal cortices" by Sagar et al., describes a study where three participants are scanned with fMRI during perception of an unusually large set of odor stimuli (n=160). The research is well-conceived and novel, and the manuscript is well written. A particularly interesting feature of this experiment is that the authors train a model on responses to aversive odors and then test it on pleasant odors, to see whether the valence code or salience code is preserved. There are also several issues that should be considered, they are listed below.

Major:

This "small-n" method leads the authors to focus on a detailed analysis within a person, rather than having less data for more people. More researchers should adopt the "small-n" strategy. However, the use of traditional group-level F statistics to investigate deviations from zero-level correlations seems inappropriate since it is still based on assumptions of generalizability to a population of participants from only three cases – why not present each individual subject result, and discuss it on a person-by-person basis?

The primary pleasantness scale is described as ranging "from appetitive to aversive", but this neglects the edibility dimension which is prominent in both odor perception and odor language (e.g. ratings may show flowers smell better but less edible than vegetables). Bad smelling odors are rarely edible, but many pleasant odors score low on the edibility dimension.

The saliency dimension is instead highlighted by the authors as a secondary feature of the pleasantness dimension (second to valence), corresponding to the magnitude of the response. Reading the introduction, the reader might question whether salience is distinct from both perceived odor intensity and valence (i.e. is the magnitude of the emotional response different from the strength and valence of the odor)? In the results, it becomes clear that salience is operationalized as the absolute valence (very pleasant or very unpleasant odors have high salience), but this should be made clear already in the introduction.

When proposing the notion that positive and negative odors are processed in a ”discontinuous” way, please specify and discuss briefly which animal models were used in the literature cited. There is no a priori reason to assume the olfactory systems of different animal species would be similar in this regard. Indeed, this is part of the reason why it is interesting to study in humans rather than just in fruit flies or mice. The sparse introduction of the core concept makes the main hypothis seem less well-founded when it appears later in the discussion.

The unexplained generalization across animal models is also present in the discussion (refs 29-31) and needs to be revised.

The literature is well covered by the authors, but some of the earlier work by Bensafi and colleagues (e.g. Fournel et al., 2016, HBM) and Li and colleagues (e.g. Krusemark et al., 2013, JNeuro) might also be relevant, especially since those authors mapped perceptual and emotional features to human fMRI pattern similarities.

What were the exact subjective rating dimensions collected on these odors, were there any more ratings done on the stimuli, apart from valence? The authors say “several perceptual descriptors, including pleasantness” with a reference to extended data table and figure, but these descriptors are not listed there. This should be revised. Besides the aforementioned edibiblity feature, intensity should be considered in olfactory perceptual research. Furthermore, olfactory perceptual neuroscience work often disregards the role of familiarity of odors, even though it is known to have a strong impact on perception. Including such dimensions would strengthen the current research.

On a more general note, the methodological approach used here appears to be based on the assumption that functionally homogeneous neurons are clustered at the resolution level of the fMRI voxel (because if there would instead be a large number of such neuronal clusters randomly distributed within each voxel, would they not average out and no signal would be detected?). Or is it rather the vascularization of the brain that operates on the scale of the voxels to enable the results? This broad methodological justification might be discussed if deemed relevant.

Minor:

Results: Please use the format ”p<0.001” or similar, as the p-value is never =0.

Discussion: ”olfactory cortices contain separate processing streams for the pleasantness of appetitive and aversive odors”. A ”processing stream” metaphor assumes a flow of information across several cortical sites. How this stream is conceived by the authors is unclear (it seems to be used as a synonym for representation), but it could be specified using available structural and functional connectivity data. This will help integrate the findings into a more cohesive package.

Reviewer #3

(Remarks to the Author)
Review of Manuscript NCOMMS-25-01167-T

This manuscript investigates the representation of odor pleasantness in the human brain based on extensive testing of three subjects with 160 different odors.
The authors conceptualize pleasantness as a two-dimensional construct of valence and salience, that are each perceptually represented as linear dimensions. They hypothesize that valence is initially cortically processed as two separate dimensions of positive and negative directionality that are only integrated into a shared representation at later processing stages. Using representational similarity analysis, they first investigate the separability of positive and negative valence representations in olfactory cortex, and subsequently demonstrate their integration into a shared pleasantness code.
The results confirm the authors' hypothesis, adding substantially to current knowledge on odor representation in the human brain. Given the novelty and timeliness of these findings, I expect this paper to generate the broad level of interest appropriate for this journal.

That being said, I have identified a number of points that the authors should address prior to publication.

Major:

- The authors present the idea of pleasantness as a combination of a linear valence dimension and a linear salience dimension as the prevailing model in psychological science. This appears to be a simplification of the state of the field where, in fact, bidirectional models of pleasurable experiences have frequently been proposed previously in behavioral research (see e.g. Schimmack, 2001). Also, the definition of salience as the second dimension seems to lack the universality that is suggested; “arousal” or “motivational strength” are alternative descriptors more commonly used. In fact, “behavioral relevance”, as the authors describe salience, seems like a problematic term as one might imagine stimuli that are highly

arousing but behaviorally irrelevant, and vice versa, and it is unclear where those would fall on a salience scale. The introduction would benefit from acknowledging these different schools of thought and outlining more clearly the motivation behind the adoption of the current framework and how this work fits in with these prior theories.
- The authors highlight the fact that their data split neatly into a positively and a negatively valenced half as a strength of their analytical approach. Inspection of the individual subjects' data, however, seems to indicate that this split does not appropriately reflect the perceptual experience within individuals; the distribution in S1 shows a shift towards the positive half of the scale, while the opposite is true for S3 (Extended Data Fig. 1). This difference appears to be reflected in the tuning maps presented in figure 4c, where clear differences in number of voxels tuned to a specific valence direction are visible between subjects, indicating they may contribute difference parts of the valence spectrum to the analyses reported here (unclear if subjects are presented in order S1, S2, S3-this should be added). The authors should critically discuss implications of these between-subject differences in affective experience for the interpretation of their findings.
- Related to this point, the authors should describe how the subjects were instructed to rate pleasantness. Was the scale anchored to a neutral midpoint? This seems to be necessary to derive a value of absolute pleasantness depending on the midpoint of the scale; otherwise, it is unclear whether the differences between subjects reflect a response bias or a true difference in directionality of experienced pleasantness. The task design in figure 1b shows a "wedge" superimposed on the scale which seems to prime participants to a linear rather than a bimodal relationship-but it also seems to ask about intensity, which is not part of the data investigated here. The authors should revise the figure and the description in the text to clarify the question and clearly describe the anchors used for the end- and midpoints.
- The authors state that analyses were "based on a previously published dataset". Are they referring to another dataset of 3 subjects with identical experimental parameters, or do the authors mean that this is another analysis on the same previously published dataset? If this is indeed the same dataset, there seems to be circularity in the definition of the ROIs, the implications of which should be addressed. Understanding is further complicated by the fact that the authors are using two indices (reference 24 and 39) to apparently refer to the same reference. Reference 42 also seems to refer to the repository for the same dataset, but again this is not clearly stated.
- Given the small number of subjects, the authors need to justify their choice of using t-tests to demonstrate the statistical significance in the SVR results; the figures clearly show that the data is unlikely to be meeting the underlying assumption of normal distribution, as a number of significant effects appear to be driven by one subject alone. Two-tailed permutation tests that, for example, shuffle the bins each trial is assigned to) could be considered instead.

Minor:
- The authors state that pleasantness was rated from -10 to +10, were these 21 discrete steps or a continuous scale? Why did the authors decide to collapse the behavioral and RSA data into 7 bins as opposed to a different number? For the imaging data, what summary statistic was used to derive a pattern for each bin? (e.g. means of betas, T-values etc.) The authors should add a reflection on the impact of the binning on their results.
- The authors should review their figure legend to make sure that each figure has all the necessary information on each parameter. Most figures lack some aspect of the figure legend that is necessary for interpretation.
- For the GLMsingle analysis, the authors should state clearly whether session indices were used. Given that the experiments were conducted over several sessions (12 sessions × 4 runs), the documentation indicates that session indices be used due to internal z-scoring within sessions.

Schimmack, U. (2001). Pleasure, displeasure, and mixed feelings: Are semantic opposites mutually exclusive? Cognition and Emotion, 15(1), 81–97. https://doi.org/10.1080/02699930126097

Reviewer #4

(Remarks to the Author)
I co-reviewed this manuscript with one of the reviewers who provided the listed reports. This is part of the Nature Communications initiative to facilitate training in peer review and to provide appropriate recognition for Early Career Researchers who co-review manuscripts.

Version 1:

Reviewer comments:

Reviewer #1

(Remarks to the Author)
I appreciate the authors' efforts in revising the manuscript and providing additional analyses that directly respond to my earlier comments. With this in mind, I have a few remaining points that I believe should be addressed. First, I think it is scientifically important to include the null results of voxel-wise RSA. Second, Extended Data Figure 5: "only absolute values defined on pleasantness (i.e. salience) is robustly represented in the brain", can you repeat the analysis creating a scale from Cool to Warm? I think it will be more accurate comparison to bipolar scale such as unpleasant-pleasant. Finally, the calculation of the pleasantness ratings still requires clarification. Each odor was presented 27–30 times, and if for each trial one of the 18 descriptors was selected, this would imply that pleasantness was rated only twice per odor before averaging.

Is this correct? Additionally, were there odors for which only a single repetition was available?

Reviewer #2

(Remarks to the Author)
In this revised manuscript, the authors have thoroughly addressed the comments provided before. A couple of minor clarifications would further enhance the readability of the manuscript text, as suggested below:

17: change from "these representations are separable in piriform cortex and amygdala" to "these pleasantness representations are separable for appetitive and aversive odors in piriform cortex and amygdala"

260: change from "importance" to "salience"

Reviewer #3

(Remarks to the Author)
We thank the authors for their thoughtful responses to our concerns. Especially the in-depth reanalysis and control analyses for the issue raised concerning the voxelwise RSA are appreciated and address our concerns fully.
However, we still have a few concerns with the manuscript as it is. These relate to the following:

Point 1:
The additional explanations provided in the introduction insufficiently address my concerns about the terminology and theoretical framework used. I understand and appreciate the idea that pleasantness and unpleasantness likely represent two different dimensions, and the data presented by the authors beautifully supports this conclusion and the argument that the brain represents these dimensions separately.
My concern relates to the fact that the introduction represents the used operationalizations of valence and salience as orthogonal dimensions while one is in fact merely a absolute transformation of the other. This conversion, while arguably an appropriate descriptor for salience for a directly incentive-compatible stimulus like money, seems to be an oversimplification of the concept of salience in the case of odors. Given the various object identities and resulting associations that odors may have, multiple dimensions in addition to valence strength may contribute to their salience and one would expect that a separate measure over and above valence would be required to appropriately capture this, for example, a measure of memorability of the item in question such as a n-back task.
Also, given the interdependency between valence and salience as defined here, the authors' definition of salience is in disagreement with the affective neuroscience literature views on arousal and valence (where valence is truly independent of arousal, e. g. rage would be high negative valence and high arousal while sadness would be low valence and low arousal, and both might be salient), so I would argue that the authors' concept of salience does not overlap with arousal, as stated in the revision. I would recommend to remove this explanation.
I would like to reiterate that the finding of positive and negative valence as separable dimensions being represented through distinct cortical mechanisms is in itself a very interesting contribution. Particularly, the results of the cross-valence decoding analysis implies that pleasant and unpleasant stimuli are not represented using a common currency until the OFC. Framing this finding as separate coding of valence and salience in my opinion diminishes its impact.

Point 2:
Thank you for the explanation for the exclusion of session indicators in the design matrix. However, based on the documentation for GLMsingle, multiple sessions should be accounted for in the sessionindicator input (c.f. https://glmsingle.readthedocs.io/en/latest/wiki.html; section 'Things to watch out for when applying GLMsingle', subsection 'Data from multiple scan sessions'). This is due to the fact that response amplitudes can and often do vary across sessions. To my knowledge, this does not introduce them as regressors (and therefore would introduce problems of collinearity) but rather internally z-scores the responses within sessions and, according to the documentation, likely improves estimates of cross-validation performance. Note that the documentation uses the terminology where 'sessions' refer to separate scanning days/visits, in contrast to 'runs'.

Point 3:
While reviewing the details concerning the methods above, I noticed that more details in the methods section of the manuscript itself would be very useful, and I would recommend that the authors consider adding those to the main manuscript in addition to their deposition of code and data to the repository. Key details that are missing are the software used, SVR parameters (number of folds, kernel, cost parameter etc.). There are also some minor inconsistencies that would benefit from further elaboration, for example the readme on the github repository says MATLAB 2023b with SPM12 and GLMsingle as necessary toolboxes, although the wrapper functions call on the svmtrain and svmpredict functions that have not been in use since MATLAB 2019 (outside of the LIBSVM toolbox).

Finally, as a very minor point–the methods section mentions that Fisher's Z-transform was applied to the SVR results. If so, are the values reported on the figures the raw correlations or the z-transformed correlations?

Reviewer #4

(Remarks to the Author)

I co-reviewed this manuscript with one of the reviewers who provided the listed reports. This is part of the Nature Communications initiative to facilitate training in peer review and to provide appropriate recognition for Early Career Researchers who co-review manuscripts.

Version 2:

Reviewer comments:

Reviewer #1

(Remarks to the Author)
In the revised manuscript, the authors have thoroughly addressed all my comments.

Reviewer #3

(Remarks to the Author)
We would like to thank the authors for the thoughtful considerations of our concerns, which have been fully addressed in this revision.

Reviewer #4

(Remarks to the Author)
I co-reviewed this manuscript with one of the reviewers who provided the listed reports. This is part of the Nature Communications initiative to facilitate training in peer review and to provide appropriate recognition for Early Career Researchers who co-review manuscripts.

Open Access This Peer Review File is licensed under a Creative Commons Attribution 4.0 International License, which permits use, sharing, adaptation, distribution and reproduction in any medium or format, as long as you give appropriate credit to the original author(s) and the source, provide a link to the Creative Commons license, and indicate if changes were made.
In cases where reviewers are anonymous, credit should be given to 'Anonymous Referee' and the source.
The images or other third party material in this Peer Review File are included in the article's Creative Commons license, unless indicated otherwise in a credit line to the material. If material is not included in the article's Creative Commons license and your intended use is not permitted by statutory regulation or exceeds the permitted use, you will need to obtain permission directly from the copyright holder.
To view a copy of this license, visit https://creativecommons.org/licenses/by/4.0/

Summary of major changes

We thank all reviewers for their constructive and thorough evaluation of our manuscript. Based on these comments, we have incorporated new analyses and modified the text throughout the manuscript.

We note that while addressing a comment by Reviewer #3, we discovered bias in our representational similarity analysis (RSA). This bias resulted from unequal numbers of trials across different pleasantness bins in our original analysis. We have now changed all RSAs to ensure they are unbiased. For this, we fixed the number of trials in each pleasantness bin to 200. While most of our initial conclusions are supported by the new analyses, this is not the case for the voxel-wise RSA. We have therefore removed all results based on the voxel-wise RSA (original Figure 4 and Figure 5). Instead, to examine coding of integrated valence and salience we now use all voxels in the ROIs. Further, to obtain more detailed results at sub-ROI level, we used anatomically defined subregions of ventral prefrontal cortex (new Figure 5). The results from these analyses show evidence for integrated representations of odor pleasantness in ventral prefrontal cortex. However, these integrated representations reflect odor salience (absolute pleasantness) rather than valence (signed pleasantness).

In addition, we conducted numerous control analyses to account for effects of sniffs; run RSAs for other perceptual features such as intensity, warm, edibility and familiarity and performed analyses in control areas such as the white matter. We also report subject-wise statistical analyses for our primary decoding results. We also repeated all decoding analyses without discretizing the pleasantness values. Taken together, these new analyses corroborate our major finding of distinguishable coding of pleasantness of appetitive and aversive odors and their integrated representation in the ventral prefrontal cortices.

Reviewer #1 (Remarks to the Author):

The study by Sagar et al. addresses a very important question in olfaction regarding the major axis of perception. In his previous paper, Sagar et al, [Nature Neuroscience 2023] showed that the encoded perceptual spaces increase in dimensionality and subjectivity from the PirC to the OFC. In the current paper the same data has been used to test how pleasantness of aversive and appetitive odors is represented in olfactory regions.

The authors used a similar modeling approach from their previous publication [Kahnt et al, 2014], however, I have a few questions regarding the use of this model in olfaction.

First, subjects rated pleasantness, why using the model and not the real pleasantness rating average?

We thank the reviewer for raising this point. Our study aimed to investigate how odor pleasantness is processed in the brain. Although subjects rated odor pleasantness on a scale that ranged from -1 (unpleasant) through 0 (neutral) to 1 (pleasant), this is not necessarily how the brain processes odor pleasantness. First, it is possible that the brain processes and represents the pleasantness of appetitive odors independently from the pleasantness of aversive ones. Second, even if the brain maps pleasant and unpleasant odor percepts onto an integrated dimension, we can think of at least two different ways in which such integration can be accomplished. First, the pleasantness of pleasant and unpleasant odors might be represented as a continuum from unpleasant (through neutral) to pleasant, similar to our pleasantness scale. Second, it is possible to collapse across unpleasant and pleasant odors and only represent the strength of the affective response, regardless of whether an odor is pleasant or unpleasant. Following a similar 'model' for these dimensions in Kahnt et al. 2014 for monetary outcomes, here we defined valence (or value) as the signed pleasantness rating, and salience as the absolute (unsigned) pleasantness. Using this model allows us to test how the brain represents pleasantness rather than presupposing it.

Second, sniffing is modulated by subjective pleasantness of an odor, and a mere sniff induce activation if olfactory areas. Although the sniff flow was treated as nuisance regressor, I am not sure this correction method is effective. I like to see figures with sniff flow vs. pleasantness ratings for each subject.

We thank the reviewer for this comment and agree that it is important to account for possible sniff-related effects. We plotted sniff airflow and volume as a function of pleasantness for each subject and found a positive correlation in two subjects, confirming that pleasantness modulated sniffing in line with previous studies[1,2]. To examine potential effects from these differences in sniff on fMRI responses, we conducted a control analysis (Extended Data Figure 2) in which we regressed out sniff-related information. Specifically, we first used the Breathmetrics toolbox[3] to compute trial-wise sniff features, such as sniff flow, volume, duration, timing, pauses etc. We then averaged each sniff feature across trials within each pleasantness bin. We then constructed pairwise correlations across sniff features for each bin pair. We then treated the resulting sniff similarity matrix as a nuisance regressor in the regression model for the RSA. This approach yielded similar results as before, showing that sniff differences did not account for our findings.

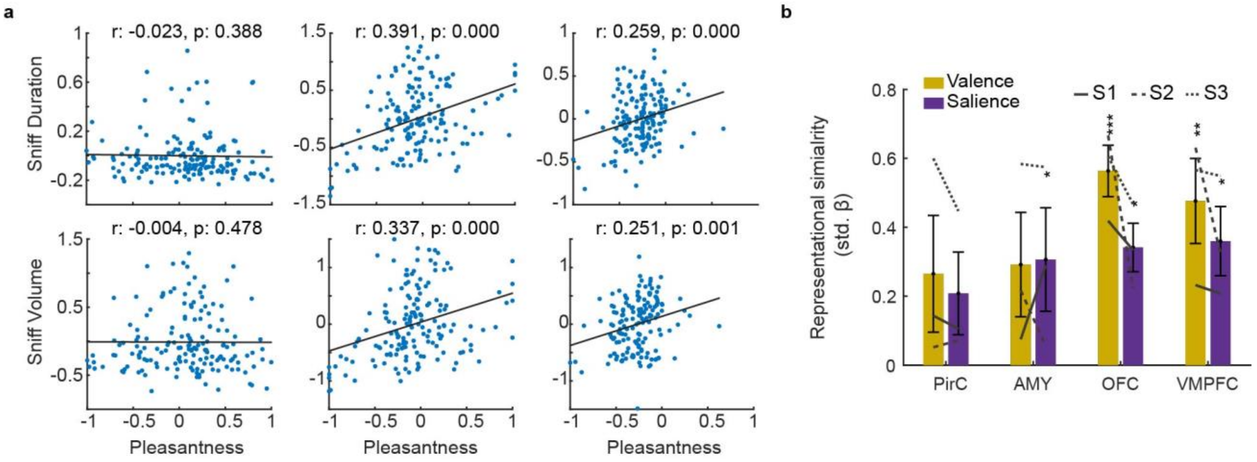

Extended Data Figure 2. Control representational similarity analysis to account for sniffing related effects. **a**. (Top) Sniff duration plotted as a function of pleasantness for 3 subjects. Data points correspond to odors (averaged across trials). Sniff Duration is positively correlated with pleasantness in S2 and S3 but not S1. **a**. (Bottom) Sniff volume plotted as a function of pleasantness for 3 subjects. Data points correspond to odors (averaged across trials). Sniff volume is positively correlated with pleasantness in S2 and S3 but not S1. **b**. Representational similarity analysis controlling for sniff related effects. A bin-by-bin sniff correlation matrix was constructed and added as a nuisance regressor in the linear model. Effect of valence is significant in OFC and VMPFC, and salience is significant in AMY, OFC and VMPFC (valence: PirC, β=0.268, p=0.170; AMY, β=0.291, p=0.124; OFC, β=0.564, p<0.001; VMPFC, β=0.480, p=0.003; salience: PirC, β=0.206, p=0.171; AMY, β=0.309, p=0.039; OFC, β=0.339, p=0.019; VMPFC, β=0.361, p=0.007; one-tailed permutation test). Data are presented as mean values and error bars indicate standard error of the mean. Asterisks denote *, p<0.05, **, p<0.01 and ***, p<0.001, respectively.

Third, the correlations values are low, for a control, please repeat the analysis with intensity / familiarity / cool-warm (assuming similar gaussian distribution as in Extended Data Figure 1).

Although intensity and cool/warm followed different distributions, we examined the correlation between pleasantness and all available perceptual descriptors (**Extended Data Figure 5a**). While familiarity and edibility were correlated with pleasantness (familiarity: r=0.41; edibility: r = 0.62), intensity and warmth were not (Intensity: r=-0.07; warm: r = 0.095). As suggested, we performed RSAs for these features, revealing significant effects for some values in some brain regions, suggesting that these perceptual features are represented in the brain areas, as shown in our previous work[4]. However, unlike pleasantness, the absolute values of these features did not reveal reliable effects in these areas. This shows that while it may be possible to substitute a different pleasantness-related perceptual feature to compute valence, only absolute values defined on pleasantness (i.e. salience) is robustly represented in the brain.

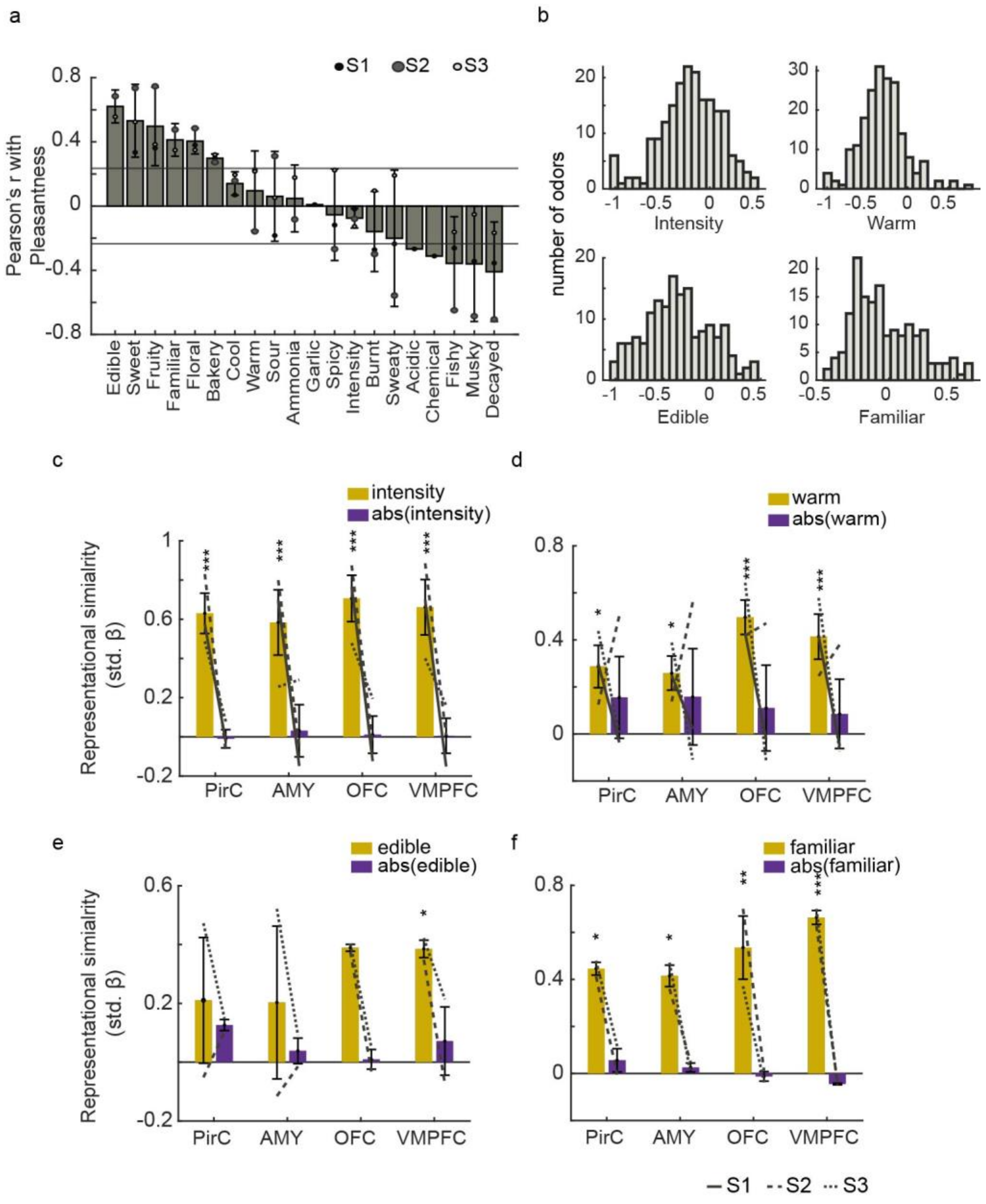

Extended Data Figure 5: Representational similarity analysis for perceptual features other than pleasantness. **a**. Pearson's correlation of perceptual ratings with pleasantness. Solid lines indicate significant threshold at p<0.05, FWE corrected, two-tailed t-test. **b**. Histograms of ratings of intensity, warm, edible and familiar perceptual features across odors **c.** Representational similarity analyses performed for intensity and absolute intensity. Standardized regression coefficient is significant for intensity but not absolute intensity in all areas. (Intensity: PirC, β=0.630, p<0.001; AMY, β=0.585, p<0.001; OFC, β=0.709, p<0.001; VMPFC, β=0.659, p<0.001; absolute Intensity: PirC, β=-0.007, p=0.728; AMY, β=0.032, p=0.583; OFC, β=0.010, p=0.762; VMPFC, β=0.005, p=0.718; one-tailed permutation test). **d**. Representational similarity analyses for warm and absolute warm. Standardized regression coefficient is significant for warm but not absolute warm in all areas. (warm: PirC, β=0.293, p=0.018; AMY, β=0.256, p=0.044; OFC, β=0.501, p<0.001; VMPFC, β=0.416, p=0.001; absolute warm: PirC, β=0.154, p=0.154; AMY, β=0.157, p=0.157; OFC, β=0.112, p=0.112; VMPFC, β=0.083, p=0.083; one-tailed permutation test). **e.** Representational similarity analyses for edibility and absolute value of edibility. Standardized regression coefficient is not significant for edible or absolute edible in all areas except for edibility in VMPFC and trending in OFC. (edible: PirC, β=0.214, p=0. 332; AMY, β=0.207, p=0. 320; OFC, β=0.389, p=0.057; VMPFC, β=0.383, p=0. 0.047; absolute edible: PirC, β=0.125, p=0.204; AMY, β=0.035, p=0.419; OFC, β=0.009, p= 0.484; VMPFC, β=0.078, p=0.284; one-tailed permutation test). **f.** Representational

similarity analyses familiarity and absolute value of familiarity. Standardized regression coefficient is significant for familiarity but not absolute familiarity in all areas. (Familiar: PirC, β=0.442, p=0. 017; AMY, β=0.410, p=0. 028; OFC, β=0.533, p=0.002; VMPFC, β=0.661, p=0. 0.000; absolute familiar: PirC, β=0.052, p=0.540; AMY, β=0.019, p=0.628; OFC, β=-0.011, p= 0.812; VMPFC, β=-0.051, p=0.873; one-tailed permutation test). Data are presented as mean values and error bars indicate standard error of the mean. Asterisks denote *, p<0.05, **, p<0.01 and ***, p<0.001, respectively.

Finally, regarding the selected ROIs, other relevant regions that should be addressed are the insula, hippocampus, and medial prefrontal cortex, as well as auditory cortex (A1) and white matter (to rule out statistical bias in the analysis). A different approach will be searchlight analysis (as in Kahnt et al 2014).

We repeated the RSA and decoding analyses in the suggested areas. We found significant effects of valence or salience in several regions, including hippocampus, insula and A1, but not in the white matter. Our imaging field of view did not cover parts of dorsal medial pFC, preventing us from conducting our analyses in dorsal mPFC (note that ventral MPFC was included in the original analyses). Of note, our goal was not to map out all brain regions in which valence or salience are represented, but to identify the nature of pleasantness coding in the key olfactory areas. We have now mentioned this in the discussion.

Discussion

We also found valence and salience-related signals outside our primary regions of interest, in line with previous work[5,6] suggesting that affective information is widely distributed across the brain[7].

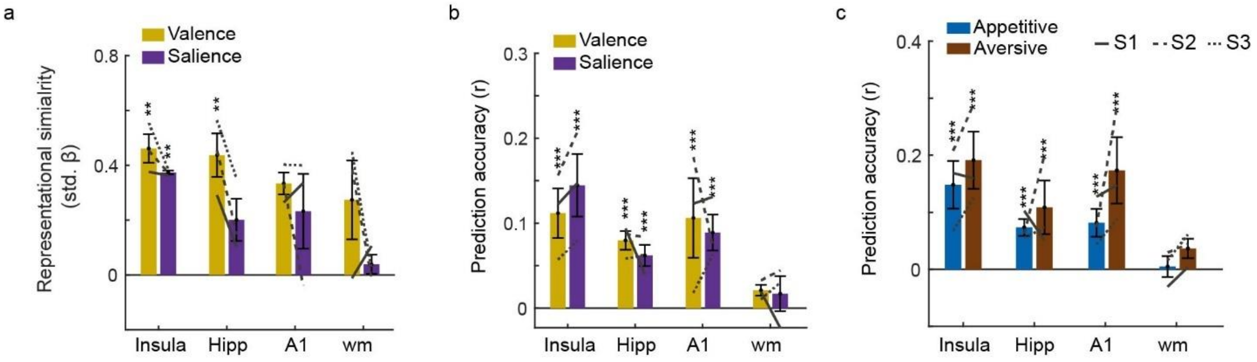

Extended Data Figure 4. Effects of valence and salience in other ROIs. **a**. Standardized regression coefficients (beta estimates) for valence and salience from a linear model predicting the neural RSM. The coefficients for valence and salience are significant in Insula, for valence in hippocampus but not elsewhere. (valence: Insula, β=0.461, p=0.002; Hippocampus, β=0.437, p=0.001; A1, β=0.334, p=0.058; wm, β=0.274, p=0.080; salience: Insula, β=0.374, p=0.003; Hippocampus, β=0.201, p=0.130; A1, β=0.232, p=0.077; wm, β=0.039, p=0.560; one-tailed permutation test). **b.** Prediction accuracy (Pearson's correlation between predicted and actual pleasantness in held out test sets) from an SVR model to predict valence and salience. Prediction accuracy is significant in all brain regions except the white matter. (valence: Insula, r=0.112, p<0.001; Hipp, r=0.080, p<0.001; A1, r=0.106, p<0.001; wm, r=0.021, p=0.165; salience: Insula, r=0.145, p<0.001; Hipp, r=0.062, p<0.001; A1, r=0.089, p<0.001; wm, r=0.017, p=0.259; N = 4400; two-tailed t-test).**c.** Prediction accuracy (Pearson's correlation between predicted and actual pleasantness in held out test sets) from an SVR model to predict pleasantness of appetitive (or aversive) odors from appetitive (or aversive) odor trials. Prediction accuracy is significant in all brain regions except the white matter. (Appetitive valence: Insula, r=0.148, p<0.001; Hipp, r=0.074, p=0.001; A1, r=0.082, p<0.001; wm, r=0.005, p=0.816; N = 2168; Aversive valence: Insula, r=0.191, p<0.001; Hipp, r=0.109, p<0.001; A1, r=0.173, p<0.001; wm, r=0.037, p=0.084; N = 2177; two-tailed t-test). Data are presented as mean values and error bars indicate standard error of the mean. Asterisks denote *, p<0.05, **, p<0.01 and ***, p<0.001, respectively.

typo: "In P0 voxels, prediction accuracy was significant for both and appetitive odors in the OFC" " but not in PirC and AMY"

Thank you. This line has been removed from the manuscript.

Reviewer #2 (Remarks to the Author):

The manuscript "Separable and integrated pleasantness coding for appetitive and aversive odors across olfactory and ventral prefrontal cortices" by Sagar et al., describes a study where three participants are scanned with fMRI during perception of an unusually large set of odor stimuli (n=160). The research is well-conceived and novel, and the manuscript is well written. A particularly interesting feature of this experiment is that the authors train a model on responses to aversive odors and then test it on pleasant odors, to see whether the valence code or salience code is preserved. There are also several issues that should be considered, they are listed below.

We thank the reviewer for their positive and constructive comments. In response to these comments, we added new analyses and revised several sections throughout the manuscript.

Major:

This "small-n" method leads the authors to focus on a detailed analysis within a person, rather than having less data for more people. More researchers should adopt the "small-n" strategy. However, the use of traditional group-level F statistics to investigate deviations from zero-level correlations seems inappropriate since it is still based on assumptions of generalizability to a population of participants from only three cases – why not present each individual subject result, and discuss it on a person-by-person basis?

We thank the reviewer for highlighting this point and giving us the opportunity to clarify. We performed all statistical analyses at the single-subject level prior to aggregating results across participants. The individual subject-wise results are now provided separately in Extended Table S2 and S3. The results were averaged for illustrative purposes in the figures. We have also removed all results based on traditional group level F statistics.

The primary pleasantness scale is described as ranging "from appetitive to aversive", but this neglects the edibility dimension which is prominent in both odor perception and odor language (e.g. ratings may show flowers smell better but less edible than vegetables). Bad smelling odors are rarely edible, but many pleasant odors score low on the edibility dimension.

We thank the reviewer for raising this point of potential confusion. We used the term "appetitive" to mean "pleasant" or "positively valanced", motivating approach behaviors, rather than "edible" or "appetizing". Thus, "appetitive" encompass both edible and non-edible pleasant odors. Accordingly, edibility ratings were correlated with pleasantness ratings, but this correlation was not perfect ($r = 0.55$ (S2) and $r = 0.68$ (S3), $p<0.001$, t-test). We clarified this in the manuscript. Of note, while edibility was represented in VMPFC, absolute edibility was not encoded in any region, revealing that our results are not driven exclusively by edibility.

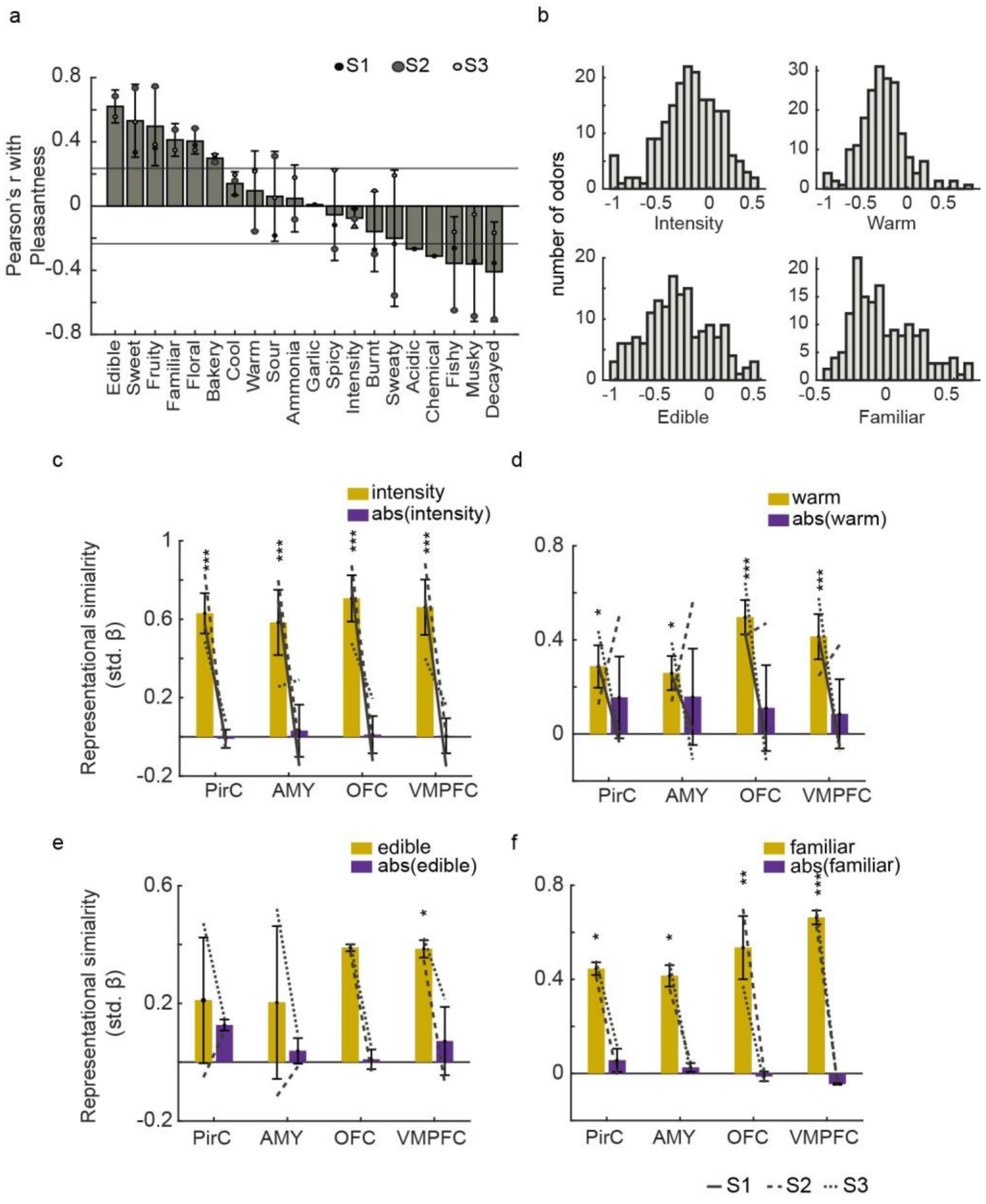

Extended Data Figure 5: Representational similarity analysis for perceptual features other than pleasantness. **a**. Pearson's correlation of perceptual ratings with pleasantness. Solid lines indicate significant threshold at p<0.05, FWE corrected, two-tailed t-test. **b**. Histograms of ratings of intensity, warm, edible and familiar perceptual features across odors **c.** Representational similarity analyses performed for intensity and absolute intensity. Standardized regression coefficient is significant for intensity but not absolute intensity in all areas. (Intensity: PirC, β=0.630, p<0.001; AMY, β=0.585, p<0.001; OFC, β=0.709, p<0.001; VMPFC, β=0.659, p<0.001; absolute Intensity: PirC, β=-0.007, p=0.728; AMY, β=0.032, p=0.583; OFC, β=0.010, p=0.762; VMPFC, β=0.005, p=0.718; one-tailed permutation test). **d**. Representational similarity analyses for warm and absolute warm. Standardized regression coefficient is significant for warm but not absolute warm in all areas. (warm: PirC, β=0.293, p=0.018; AMY, β=0.256, p=0.044; OFC, β=0.501, p<0.001; VMPFC, β=0.416, p=0.001; absolute warm: PirC, β=0.154, p=0.154; AMY, β=0.157, p=0.157; OFC, β=0.112, p=0.112; VMPFC, β=0.083, p=0.083; one-tailed permutation test). **e.** Representational similarity analyses for edibility and absolute value of edibility. Standardized regression coefficient is not significant for edible or absolute edible in all areas except for edibility in VMPFC and trending in OFC. (edible: PirC, β=0.214, p=0. 332; AMY, β=0.207, p=0. 320; OFC, β=0.389, p=0.057; VMPFC, β=0.383, p=0. 0.047; absolute edible: PirC, β=0.125, p=0.204; AMY, β=0.035, p=0.419; OFC, β=0.009, p= 0.484; VMPFC, β=0.078, p=0.284; one-tailed permutation test). **f.** Representational similarity analyses familiarity and absolute value of familiarity. Standardized regression coefficient is significant for

familiarity but not absolute familiarity in all areas. (Familiar: PirC, β=0.442, p=0. 017; AMY, β=0.410, p=0. 028; OFC, β=0.533, p=0.002; VMPFC, β=0.661, p=0. 0.000; absolute familiar: PirC, β=0.052, p=0.540; AMY, β=0.019, p=0.628; OFC, β=-0.011, p= 0.812; VMPFC, β=-0.051, p=0.873; one-tailed permutation test). Data are presented as mean values and error bars indicate standard error of the mean. Asterisks denote *, $p<0.05$, **, $p<0.01$ and ***, $p<0.001$, respectively.

The saliency dimension is instead highlighted by the authors as a secondary feature of the pleasantness dimension (second to valence), corresponding to the magnitude of the response. Reading the introduction, the reader might question whether salience is distinct from both perceived odor intensity and valence (i.e. is the magnitude of the emotional response different from the strength and valence of the odor)? In the results, it becomes clear that salience is operationalized as the absolute valence (very pleasant or very unpleasant odors have high salience), but this should be made clear already in the introduction.

We have now clarified in the introduction that we defined odor salience specifically as the absolute pleasantness (i.e., strongly positive or negative). Further, to explore the link between intensity and salience, we correlated intensity ratings with absolute pleasantness ratings (salience). Indeed, there is a positive correlation between absolute pleasantness and intensity (avg $r = 0.30$, $p < 0.001$, $N = 160$ odors, two-tailed t-test). However, controlling for intensity provides qualitatively similar results as the original analysis (Extended Data Figure 6), suggesting that the correlation between intensity and salience does not explain our decoding results.

Introduction

Whereas valence is directly based on pleasantness and thought to drive approach or avoidance responses [8,9], salience is operationalized as absolute pleasantness (very pleasant or very unpleasant odors have high salience) and relates to the magnitude of the affective response [10].

When proposing the notion that positive and negative odors are processed in a ”discontinuous” way, please specify and discuss briefly which animal models were used in the literature cited. There is no a priori reason to assume the olfactory systems of different animal species would be similar in this regard. Indeed, this is part of the reason why it is interesting to study in humans rather than just in fruit flies or mice. The sparse introduction of the core concept makes the main hypothis seem less well-founded when it appears later in the discussion.
The unexplained generalization across animal models is also present in the discussion (refs 29-31) and needs to be revised.

We have now specified in the Introduction and discussion which studies were conducted in rodents versus in humans. We also now acknowledge in the discussion that direct parallels between human and animal models of olfaction remain only partly inferred.

Introduction

Indeed, work across species suggests that the brain may separately process the pleasantness of appetitive and aversive odors. Specifically, distinguishable coding of positive and negative odor valence has been reported in olfactory areas such as the olfactory bulb, olfactory tubercle, amygdala, piriform cortex, and elsewhere in the brain of flies[11], rodents[11-13] and humans[14-16].

…

Human studies in other sensory modalities suggest that the brain represents such continuous dimensions of valence and salience in cortical areas [10,17,18]. For instance, activity in ventromedial prefrontal cortex (VMPFC) correlates with the value of both appetitive and aversive stimuli [19], and response patterns in orbitofrontal cortex (OFC) represent value across cues that predict positive and negative monetary outcomes [10]. While this implies ventral prefrontal regions as key candidates for integrating pleasantness information across valence domains into continuous dimensions, studies in rodents [20,21] [22] and humans[9] have shown similar signals in subcortical structures.

Discussion

While several studies have examined odor valence coding in humans and animals, parallels between species need to be inferred. Work in humans suggests that odor pleasantness is transmitted from the olfactory bulb to the PirC [15], but given the diffuse and overlapping input into PirC [23,24], as well as its general association cortex-like architecture [25,26] as known from rodent studies, the valence-based segregation in PirC may also be generated locally or imposed by top down input.

The literature is well covered by the authors, but some of the earlier work by Bensafi and colleagues (e.g. Fournel et al., 2016, HBM) and Li and colleagues (e.g. Krusemark et al., 2013, JNeuro) might also be relevant, especially since those authors mapped perceptual and emotional features to human fMRI pattern similarities.

Thank you for suggesting these references – we have now added the citations to the text.

Introduction:

As such, it is correlated with activity in olfactory brain areas, including piriform cortex, amygdala, and orbitofrontal cortex [2-12].

What were the exact subjective rating dimensions collected on these odors, were there any more ratings done on the stimuli, apart from valence? The authors say "several perceptual descriptors, including pleasantness" with a reference to extended data table and figure, but these descriptors are not listed there. This should be revised. Besides the aforementioned edibiblity feature, intensity should be considered in olfactory perceptual research. Furthermore, olfactory perceptual neuroscience work often disregards the role of familiarity of odors, even though it is known to have a strong impact on perception. Including such dimensions would strengthen the current research.

We thank the reviewer for this comment. Subjects rated each odor on 18 perceptual descriptors. All subjects rated pleasantness, intensity, fishy, burnt, sour, decayed, musky, fruity, sweaty, cool, floral, sweet, warm, bakery, and, spicy. In addition to these descriptors, S1 rated acidic, garlic and chemical-like, whereas S2 and S3 rated ammonia, edibility and familiarity. These descriptors are now listed in the Methods section.

Our previous publication using these data focused extensively on neural representations of these perceptual dimensions. In contrast, the current manuscript focuses on how the brain processes the pleasantness of appetitive and aversive odors.

However, we repeated the current analysis with several perceptual descriptors that are related to valence and salience: warm, edible, familiar and intensity. While some of these descriptors were represented in several brain areas (as shown in our previous work), their absolute magnitudes were not, suggesting that while it may be possible to substitute a different pleasantness-related perceptual feature to compute valence, only salience defined based on pleasantness is represented in the brain (see **Extended Data Figure 5,** reproduced above).

On a more general note, the methodological approach used here appears to be based on the assumption that functionally homogeneous neurons are clustered at the resolution level of the fMRI voxel (because if there would instead be a large number of such neuronal clusters randomly distributed within each voxel, would they not average out and no signal would be detected?). Or is it rather the vascularization of the brain that operates on the scale of the voxels to enable the results? This broad methodological justification might be discussed if deemed relevant.
We thank the reviewer for raising this important point which is relevant for all MVPA approaches. The clustering of functionally homogeneous neurons likely occurs on a finer scale than the resolution of voxels. However, this does not mean that different populations cancel out entirely at the level of voxels. For instance, random sampling of functionally homogeneous neuronal populations by individual voxels will induce small sampling biases, such that information can be recovered from the pattern of these biases across voxels[30,31]. Moreover, such voxel-wise biases are likely further amplified by structure in the vasculature which may act as a complex spatiotemporal filter.[32,33] We have now added some discussion on how clustering of functional tuning at the level of neuronal populations could drive information in multi-voxel activity patterns.

Discussion:
Of note, our analyses examined neural representations of pleasantness in multi-voxel activity patterns. The size of a fMRI voxel almost certainly exceeds the size of neuronal populations with homogenous coding schemes. Thus, the information sampled by our approach likely results from a combination of factors, including clustering of neuronal populations with similar tuning, biased sampling of neuronal populations at level of individual voxels[30,31], and filtering properties of the vasculature[32,33].

Minor:

Results: Please use the format ”p<0.001” or similar, as the p-value is never =0.
We have modified the notation throughout the manuscript.

Discussion: ”olfactory cortices contain separate processing streams for the pleasantness of appetitive and aversive odors”. A ”processing stream” metaphor assumes a flow of information across several cortical sites. How this stream is conceived by the authors is unclear (it seems to be used as a synonym for representation), but it could be specified using available structural and functional connectivity data. This will help integrate the findings into a more cohesive package.

Thank you for raising this point. We used the term “processing stream” to indicate the idea that the brain sequentially processes information about odors across several stages. However, the reviewer is correct that our analyses do not focus on such sequential information processing across areas but rather infer this from brain anatomy and observing different information in different brain areas. We have revised the manuscript accordingly and replaced processing stream with representation.

Reviewer #3 (Remarks to the Author):

Review of Manuscript NCOMMS-25-01167-T

This manuscript investigates the representation of odor pleasantness in the human brain based on extensive testing of three subjects with 160 different odors.

The authors conceptualize pleasantness as a two-dimensional construct of valence and salience, that are each perceptually represented as linear dimensions. They hypothesize that valence is initially cortically processed as two separate dimensions of positive and negative directionality that are only integrated into a shared representation at later processing stages. Using representational similarity analysis, they first investigate the separability of positive and negative valence representations in olfactory cortex, and subsequently demonstrate their integration into a shared pleasantness code.

The results confirm the authors' hypothesis, adding substantially to current knowledge on odor representation in the human brain. Given the novelty and timeliness of these findings, I expect this paper to generate the broad level of interest appropriate for this journal.

That being said, I have identified a number of points that the authors should address prior to publication.

We thank the reviewer for these positive comments and for making valuable suggestions to improve the manuscript. In response, we have added new analyses and made text revisions throughout the manuscript.

Major:

The authors present the idea of pleasantness as a combination of a linear valence dimension and a linear salience dimension as the prevailing model in psychological science. This appears to be a simplification of the state of the field where, in fact, bidirectional models of pleasurable experiences have frequently been proposed previously in behavioral research (see e.g. Schimmack, 2001). Also, the definition of salience as the second dimension seems to lack the universality that is suggested; "arousal" or "motivational strength" are alternative descriptors more commonly used. In fact, "behavioral relevance", as the authors describe salience, seems like a problematic term as one might imagine stimuli that are highly arousing but behaviorally irrelevant, and vice versa, and it is unclear where those would fall on a salience scale. The introduction would benefit from acknowledging these different schools of thought and outlining more clearly the motivation behind the adoption of the current framework and how this work fits in with these prior theories.

We thank the reviewer for raising this point. We fully agree with the notion that in experimental psychology, affective stimuli are often described using the two orthogonal dimensions valence and arousal. Here we used a different but related approach. Our study aimed to investigate how odor pleasantness is processed in the brain. Although subjects rated odor pleasantness on a scale that ranged from -1 (unpleasant) through 0 (neutral) to 1 (pleasant), this is not necessarily

how the brain processes odor pleasantness. First, it is possible that the brain processes and represents the pleasantness of appetitive odors independently from the (un)pleasantness of aversive odors. Second, even if the brain maps appetitive and aversive odor percepts onto an integrated dimension, we can think of at least two different ways this integration takes place. First, the pleasantness of appetitive and aversive odors might be represented as a continuum from unpleasant (through neutral) to pleasant (like our pleasantness scale). Second, it is possible to collapse across aversive and appetitive odors and only represent the strength of the affective response, regardless of whether an odor is pleasant or unpleasant. Following a similar 'model' that was used in previous work[10] for monetary outcomes, here we defined valence (or value) as the signed pleasantness rating and salience as the absolute (unsigned) pleasantness ratings. Both dimensions are important for motivated behavior. Whereas valence determines whether odors should be approached or avoided, salience determines the strength of this affective response.

We agree there is conceptual overlap between salience as defined here and arousal. However, in our model, salience is not linearly independent of valence but describes a U-shaped function of pleasantness. This definition of salience is often termed 'motivational salience' in the behavioral and decision neuroscience literature[20] and intimately connected to learning and behavior, which is why we related this concept to behavioral relevance. However, we do agree that specially for humans, a stimulus can be salient without being behaviorally relevant and have removed this term from the manuscript.

We now more clearly define valence and (motivational) salience early in the introduction and discuss how it is related to other concepts such as those that include "arousal".

Introduction

*Psychological theories often characterize affective experiences by two dimensions; valence and arousal[34,35]. Here, we adopt a related but different framework derived from behavioral and decision neuroscience, in which stimulus pleasantness (more generally, stimulus value) gives rise to two continuous dimensions, namely valence (**Fig 1a** top) and salience (**Fig 1a** bottom**)**. Whereas valence is directly based on pleasantness and thought to drive approach or avoidance responses [8,9], salience is operationalized as absolute pleasantness (very pleasant or very unpleasant odors have high salience) and relates to the magnitude of the affective response [10]. Note that this notion of salience overlaps with concepts like "arousal" and "motivational intensity" and emphasizes the capacity of a stimulus to evoke either positive or negative affective states.*

The authors highlight the fact that their data split neatly into a positively and a negatively valenced half as a strength of their analytical approach. Inspection of the individual subjects' data, however, seems to indicate that this split does not appropriately reflect the perceptual experience within individuals; the distribution in S1 shows a shift towards the positive half of the scale, while the opposite is true for S3 (Extended Data Fig. 1). This difference appears to be reflected in the tuning maps presented in figure 4c, where clear differences in number of voxels tuned to a specific valence direction are visible between subjects, indicating they may contribute difference parts of the valence spectrum to the analyses reported here (unclear if subjects are presented in order S1, S2, S3-this should

be added). The authors should critically discuss implications of these between-subject differences in affective experience for the interpretation of their findings.

We sincerely thank the reviewer for raising this point. In fact, this comment sent us on a journey to investigate the effects of unequal trial numbers in different pleasantness bins on the results of the RSA. We discovered that differences in this distribution biased these analyses (especially the voxel-wise RSA). This bias is effectively removed by including the same number of trials in each pleasantness bin. This also ensures that the number of odors with positive and negative pleasantness ratings are equal. Given the voxel-wise RSA was especially affected by this bias, we have removed all results based on these analyses. All analyses included in the revised manuscript are now based on unbiased RSAs.

Related to this point, the authors should describe how the subjects were instructed to rate pleasantness. Was the scale anchored to a neutral midpoint? This seems to be necessary to derive a value of absolute pleasantness depending on the midpoint of the scale; otherwise, it is unclear whether the differences between subjects reflect a response bias or a true difference in directionality of experienced pleasantness. The task design in figure 1b shows a "wedge" superimposed on the scale which seems to prime participants to a linear rather than a bimodal relationship-but it also seems to ask about intensity, which is not part of the data investigated here. The authors should revise the figure and the description in the text to clarify the question and clearly describe the anchors used for the end- and midpoints.

Participants rated pleasantness on a continuous scale from -10 (dislike) to +10 (like very much), with a defined midpoint of 0 that was labeled "Neutral." We have revised Figure 1b to more accurately reflect this scale (removing "intensity" in that schematic). We now explicitly state in the text that a rating of 0 indicated a neutral value.

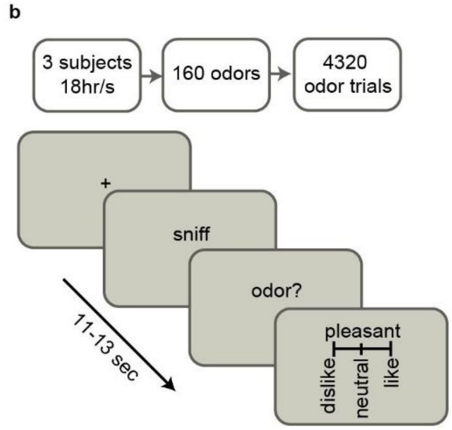

Figure 2b. Experimental design. Three human subjects were scanned for 18 hours each (4320-4560 trials) as they smelled and rated odors on perceptual features including pleasantness. Odor pleasantness was rated on a labeled scale ranging from -10=dislike very much, through 0=neutral, to 10=like very much.

The authors state that analyses were "based on a previously published dataset". Are they referring to another dataset of 3 subjects with identical experimental parameters, or do the authors mean that this is another analysis on the same previously published dataset? If this is indeed the same dataset, there seems to be circularity in the definition of the ROIs,

the implications of which should be addressed. Understanding is further complicated by the fact that the authors are using two indices (reference 24 and 39) to apparently refer to the same reference. Reference 42 also seems to refer to the repository for the same dataset, but again this is not clearly stated.

For the current manuscript, we analyzed the same previously published dataset, not a new one. However, the current analyses are non-overlapping with the previous paper which focused on neural representations of subjective olfactory percepts. We further note that our ROI masks were anatomically defined (i.e., not functionally defined), and so there was no circularity between ROI selection and functional analyses. Specifically, we inverse-normalized masks from anatomical atlases (AAL atlas) and manually optimized them in the subject's native spaces (rather than isolating them via thresholded fMRI results). Unlike our previously published work, we did not limit the analyses to voxels with significant odor-evoked responses. We now clarify this in the methods. We have also corrected redundant references in the manuscript.

Methods:
However, unlike our previous study, analysis was based on all gray-matter voxels within each ROI.

Given the small number of subjects, the authors need to justify their choice of using t-tests to demonstrate the statistical significance in the SVR results; the figures clearly show that the data is unlikely to be meeting the underlying assumption of normal distribution, as a number of significant effects appear to be driven by one subject alone. Two-tailed permutation tests that, for example, shuffle the bins each trial is assigned to) could be considered instead.

We thank the reviewer for raising this important point and for giving us the opportunity to clarify. For the decoding analyses, we evaluated significance of prediction accuracies at the single-subject level using t-tests with degrees of freedom corresponding to the number of predictions (i.e., trials) per subject. For simplified results reporting and illustrative purposes, we also ran statistics on aggregated data. We have now clarified in the methods that all statistics were also computed at a subject-wise level and tabulated the subject-wise statistics **in Supplementary Table S2 and S3**. Applying permutation-based approaches to the trial-wise SVRs is computationally too demanding; however, we now use permutation tests in the RSA and obtain similar results as before.

Methods (decoding analysis):
*Since permutation tests were not computationally feasible for our single-trial decoding analyses, we performed subject-wise parametric tests for each ROI. Specifically, we computed the p-value for subject-wise prediction accuracies (correlation coefficients) using two-tailed t-tests, with the degrees of freedom corresponding to the number of predictions (i.e., trials) per subject. These results are summarized in **Supplementary Table S2 and S3**. For the purposes of summarizing and illustration, we also performed group-level analyses where we computed the p-value of the average Fisher's Z-transformed correlation coefficients across subjects using a two-tailed t-test with the degrees of freedom corresponding to the mean number of predictions (i.e., trials) across subjects.*

Minor:
The authors state that pleasantness was rated from -10 to +10, were these 21 discrete steps or a continuous scale? Why did the authors decide to collapse the behavioral and RSA data into 7 bins as opposed to a different number? For the imaging data, what summary statistic was used to derive a pattern for each bin? (e.g. means of betas, T-values etc.) The authors should add a reflection on the impact of the binning on their results.

The scale was continuous from -10 to +10. We discretized trial-wise pleasantness ratings into seven bins for RSA, since 7 bins optimized the trade-off between a relatively high resolution and a sufficient number of trials per bin. In practice, our results are relatively robust to variations in bin count. We obtain similar results (especially In OFC and VMPFC) when running the RSA with 5 and 9 bins. For each bin, we took the average voxel response patterns across all trials that fell within that bin (i.e., mean beta values from the single-trial GLMsingle output). We have clarified this procedure and our rationale in the methods section.

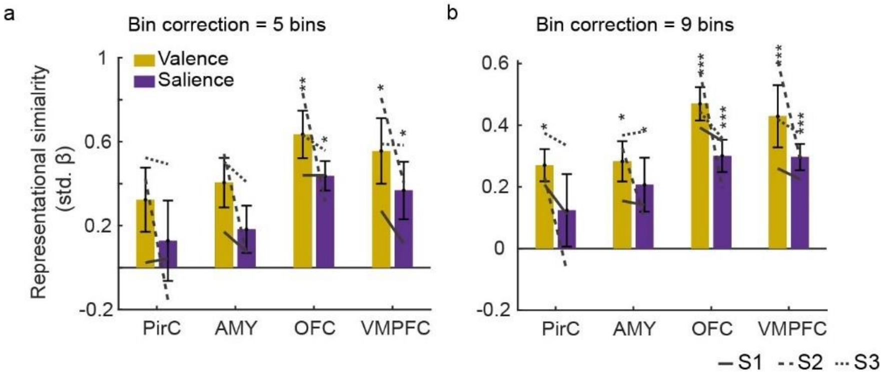

Extended Data Figure 3: Representational similarity analysis controlling for number of bins used for discretizing pleasantness. **a.** RSA when pleasantness ratings are discretized with 5 bins. The coefficients for valence and salience are significant in OFC and VMPFC and trending for valence in AMY (valence: PirC, β=0.328, p=0.141; AMY, β=0.409, p=0.063; OFC, β=0.635, p=0.003; VMPFC, β=0.561, p=0.010; salience: PirC, β=0.130, p=0.364; AMY, β=0.179, p=0.271; OFC, β=0.010, p=0.026; VMPFC, β=0.370, p=0.050; one-tailed permutation test). **b.** RSA when pleasantness ratings are discretized with 9 bins. The coefficients for valence and salience are significant in all regions except salience for PirC. (valence: PirC, β=0.270, p=0.048; AMY, β=0.283, p=0.026; OFC, β=0.470, p=0.000; VMPFC, β=0.430, p=0.000; salience: PirC, β=0.126, p=0.085; AMY, β=0.207, p=0.012; OFC, β=0.297, p=0.000 VMPFC, β=0.298, p=0.000; one-tailed permutation test). Data are presented as mean values and error bars indicate standard error of the mean. Asterisks denote *, p<0.05, **, p<0.01 and ***, p<0.001, respectively.

Methods:
Here we performed the analysis based on discretizing pleasantness in 7 bins. We chose 7 bins since it optimized the trade-off between a relatively high resolution and a sufficient numbers of trials per bin.

The authors should review their figure legend to make sure that each figure has all the necessary information on each parameter. Most figures lack some aspect of the figure legend that is necessary for interpretation.

Thank you for this comment. We reviewed each figure legend to include full information about the statistical analyses, binning, and measures used (e.g., correlation coefficients, regression betas, standardized scales).

Q: - For the GLMsingle analysis, the authors should state clearly whether session indices were used. Given that the experiments were conducted over several sessions (12 sessions × 4 runs), the documentation indicates that session indices be used due to internal z-scoring within sessions.

We used GLMsingle while accounting for run-wise baseline shifts and scaling differences, consistent with the recommended GLMsingle setting of using separate regressors per run. We did not explicitly append session-wise indices above and beyond this to minimize collinearity since two odors in distinct sessions necessarily belonged to different runs.

Schimmack, U. (2001). Pleasure, displeasure, and mixed feelings: Are semantic opposites mutually exclusive? Cognition and Emotion, 15(1), 81–97. https://doi.org/10.1080/02699930126097

We now refer to this paper in the discussion.

Discussion
Moreover, this notion of discontinuous pleasantness coding parallels previous work showing that pleasure and displeasure evoked by visual stimuli do not fall on a unidimensional axis[36].

Reviewer #4 (Remarks to the Author):

I co-reviewed this manuscript with one of the reviewers who provided the listed reports. This is part of the Nature Communications initiative to facilitate training in peer review and to provide appropriate recognition for Early Career Researchers who co-review manuscripts.

Thak you for your time and effort in reviewing our manuscript.

1 Prescott, J., Burns, J. & Frank, R. A. Influence of odor hedonics, food-relatedness, and motivational state on human sniffing. *Chemosensory perception* **3**, 85-90 (2010).

2 Ferdenzi, C., Fournel, A., Thévenet, M., Coppin, G. & Bensafi, M. Viewing olfactory affective responses through the sniff prism: effect of perceptual dimensions and age on olfactomotor responses to odors. *Frontiers in Psychology* **6**, 1776 (2015).

3 Noto, T., Zhou, G., Schuele, S., Templer, J. & Zelano, C. Automated analysis of breathing waveforms using BreathMetrics: a respiratory signal processing toolbox. *Chemical senses* **43**, 583-597 (2018).

4 Sagar, V., Shanahan, L. K., Zelano, C. M., Gottfried, J. A. & Kahnt, T. High-precision mapping reveals the structure of odor coding in the human brain. *Nat Neurosci* **26**, 1595-1602, doi:10.1038/s41593-023-01414-4 (2023).

5 Roy-Côté, F., Zahal, R., Frasnelli, J., Nguyen, D. K. & Boucher, O. Insula and olfaction: a literature review and case report. *Brain Sciences* **11**, 198 (2021).

6 Kehl, M. S. *et al.* Single-neuron representations of odours in the human brain. *Nature* **634**, 626-634 (2024).

7 Vickery, T. J., Chun, M. M. & Lee, D. Ubiquity and specificity of reinforcement signals throughout the human brain. *Neuron* **72**, 166-177 (2011).

8 Yeshurun, Y. & Sobel, N. An odor is not worth a thousand words: from multidimensional odors to unidimensional odor objects. *Annual review of psychology* **61**, 219-241 (2010).

9 Jin, J., Zelano, C., Gottfried, J. A. & Mohanty, A. Human amygdala represents the complete spectrum of subjective valence. *Journal of Neuroscience* **35**, 15145-15156 (2015).

10 Kahnt, T., Park, S. Q., Haynes, J.-D. & Tobler, P. N. Disentangling neural representations of value and salience in the human brain. *Proceedings of the National Academy of Sciences* **111**, 5000-5005 (2014).

11 Knaden, M. & Hansson, B. S. Mapping odor valence in the brain of flies and mice. *Current Opinion in Neurobiology* **24**, 34-38 (2014).

12 Howe, J. R. *et al.* Control of innate olfactory valence by segregated cortical amygdala circuits. *bioRxiv* (2024).

13 Gadziola, M. A., Tylicki, K. A., Christian, D. L. & Wesson, D. W. The olfactory tubercle encodes odor valence in behaving mice. *Journal of Neuroscience* **35**, 4515-4527 (2015).

14 Iravani, B., Schaefer, M., Wilson, D. A., Arshamian, A. & Lundström, J. N. The human olfactory bulb processes odor valence representation and cues motor avoidance behavior. *Proceedings of the National Academy of Sciences* **118**, e2101209118 (2021).

15 Nordén, F. *et al.* The human olfactory bulb communicates perceived odor valence to the piriform cortex in the gamma band and receives a refined representation back in the beta band. *PLoS biology* **22**, e3002849 (2024).

16 Grabenhorst, F., Rolls, E. T., Margot, C., Da Silva, M. A. & Velazco, M. I. How pleasant and unpleasant stimuli combine in different brain regions: odor mixtures. *Journal of Neuroscience* **27**, 13532-13540 (2007).

17 Iigaya, K. *et al.* Neural mechanisms underlying the hierarchical construction of perceived aesthetic value. *Nature Communications* **14**, 127 (2023).
18 Lee, S. A. *et al.* Brain representations of affective valence and intensity in sustained pleasure and pain. *Proceedings of the National Academy of Sciences* **121**, e2310433121 (2024).
19 Plassmann, H., O'Doherty, J. P. & Rangel, A. Appetitive and aversive goal values are encoded in the medial orbitofrontal cortex at the time of decision making. *J Neurosci* **30**, 10799-10808, doi:10.1523/JNEUROSCI.0788-10.2010 (2010).
20 Matsumoto, M. & Hikosaka, O. Two types of dopamine neuron distinctly convey positive and negative motivational signals. *Nature* **459**, 837-841 (2009).
21 Bromberg-Martin, E. S., Matsumoto, M. & Hikosaka, O. Dopamine in motivational control: rewarding, aversive, and alerting. *Neuron* **68**, 815-834 (2010).
22 Steinberg, E. E. *et al.* Amygdala-midbrain connections modulate appetitive and aversive learning. *Neuron* **106**, 1026-1043. e1029 (2020).
23 Illig, K. R. & Haberly, L. B. Odor-evoked activity is spatially distributed in piriform cortex. *Journal of Comparative Neurology* **457**, 361-373 (2003).
24 Rennaker, R. L., Chen, C.-F. F., Ruyle, A. M., Sloan, A. M. & Wilson, D. A. Spatial and temporal distribution of odorant-evoked activity in the piriform cortex. *Journal of Neuroscience* **27**, 1534-1542 (2007).
25 Johnson, D. M., Illig, K. R., Behan, M. & Haberly, L. B. New features of connectivity in piriform cortex visualized by intracellular injection of pyramidal cells suggest that "primary" olfactory cortex functions like "association" cortex in other sensory systems. *Journal of Neuroscience* **20**, 6974-6982 (2000).
26 Bekkers, J. M. & Suzuki, N. Neurons and circuits for odor processing in the piriform cortex. *Trends in neurosciences* **36**, 429-438 (2013).
27 Winston, J. S., Gottfried, J. A., Kilner, J. M. & Dolan, R. J. Integrated neural representations of odor intensity and affective valence in human amygdala. *Journal of Neuroscience* **25**, 8903-8907 (2005).
28 Krusemark, E. A., Novak, L. R., Gitelman, D. R. & Li, W. When the sense of smell meets emotion: anxiety-state-dependent olfactory processing and neural circuitry adaptation. *Journal of Neuroscience* **33**, 15324-15332 (2013).
29 Fournel, A., Ferdenzi, C., Sezille, C., Rouby, C. & Bensafi, M. Multidimensional representation of odors in the human olfactory cortex. *Human brain mapping* **37**, 2161-2172 (2016).
30 Kahnt, T. A decade of decoding reward-related fMRI signals and where we go from here. *Neuroimage* **180**, 324-333 (2018).
31 Kriegeskorte, N., Goebel, R. & Bandettini, P. Information-based functional brain mapping. *Proceedings of the National Academy of Sciences* **103**, 3863-3868 (2006).
32 Shmuel, A., Yacoub, E., Chaimow, D., Logothetis, N. K. & Ugurbil, K. Spatio-temporal point-spread function of fMRI signal in human gray matter at 7 Tesla. *Neuroimage* **35**, 539-552 (2007).
33 Gardner, J. L. Is cortical vasculature functionally organized? *Neuroimage* **49**, 1953-1956 (2010).
34 Russell, J. A. A circumplex model of affect. *Journal of personality and social psychology* **39**, 1161 (1980).
35 Kuppens, P., Tuerlinckx, F., Russell, J. A. & Barrett, L. F. The relation between valence and arousal in subjective experience. *Psychological bulletin* **139**, 917 (2013).
36 Schimmack, U. Pleasure, displeasure, and mixed feelings: Are semantic opposites mutually exclusive? *Cognition & Emotion* **15**, 81-97 (2001).

Reviewer #1 (Remarks to the Author):

I appreciate the authors' efforts in revising the manuscript and providing additional analyses that directly respond to my earlier comments. With this in mind, I have a few remaining points that I believe should be addressed.

First, I think it is scientifically important to include the null results of voxel-wise RSA.

We thank the reviewer for their thorough assessment. We have included the null results from the voxel-wise RSA in **Extended Data Figure 7**. Specifically, we plot the density plot of voxel-wise bivariate representational similarities for pleasantness of appetitive and aversive odors for all ROIs. The results show that univariate analyses performed in this way lead to null results for most voxels, with the highest density of voxels around (0,0), highlighting the need for multivariate approaches. We also describe this analysis in the methods and refer to it in the main manuscript.

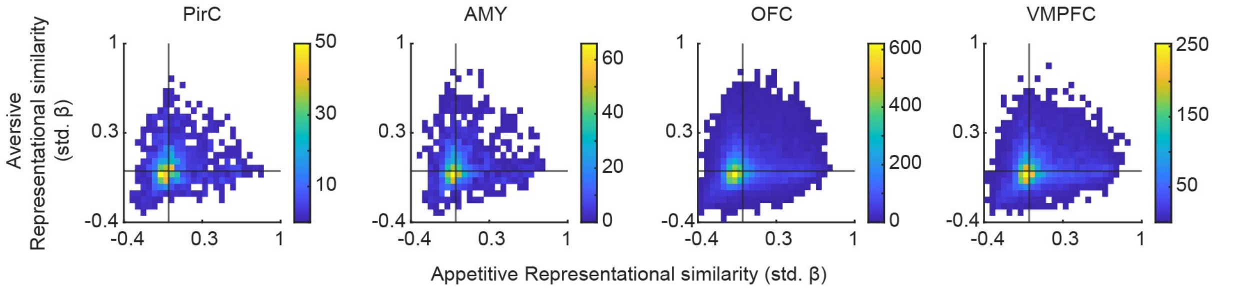

Extended Data Figure 7: Distribution of voxel-wise RSA coefficients for the pleasantness of appetitive and aversive odors. Hot colors indicate higher density of voxels. The highest density is close to the central bins (0,0).

Results:

*"We did not find significant pleasantness coding for appetitive and aversive odors using a voxel-wise univariate analysis (**Extended Data Figure 7**)".*

Methods (Representational Similarity Analyses (RSA):

"We adapted a univariate RSA to examine coding of pleasantness for appetitive and aversive odors in individual voxels. In other words, for each voxel we created a bin-by-bin neural similarity matrix by taking pairwise absolute differences in voxel responses for two bins of pleasantness (each consisting of the same number of odor trials). Then, similar to the multivariate RSA, we regressed hypothetical similarity matrices for appetitive and aversive pleasantness against the neural RSM for every voxel, resulting in a bivariate estimate of voxel-wise representation of pleasantness for appetitive and aversive odors."

Second, Extended Data Figure 5: "only absolute values defined on pleasantness (i.e. salience) is robustly represented in the brain", can you repeat the analysis creating a scale from Cool to Warm? I think it will be more accurate comparison to bipolar scale such as unpleasant-pleasant.

We thank the reviewer for suggestion. Following this suggestion, we created a new scale by taking the difference between warm and cold ratings, and repeated the RSA for signed and unsigned values of this scale. This did not reveal significant effects, except for the absolute values in OFC, suggesting that our results are indeed specific to pleasantness. The results are now included in **Extended Data Figure 5g.**

g

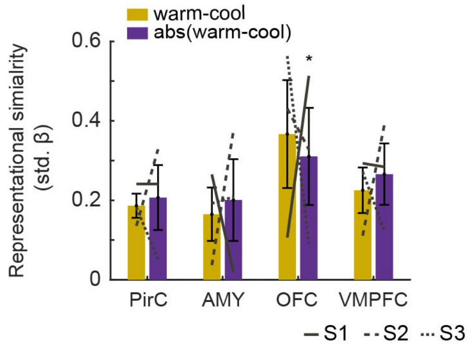

Extended Data Figure 5g. Representational similarity analyses for warm-cool and absolute values of warm-cool. Standardized regression coefficient is not significant for this composite dimension except for a weak effect for absolute warm-cool in the OFC. (warm-cool: PirC, β=0.186, p=0. 293; AMY, β=0.165, p=0. 378; OFC, β=0.366, p=0.057; VMPFC, β=0.225, p=0. 0.267; absolute warm-cool: PirC, β=0.207, p=0.108; AMY, β=0.200, p=0.128; OFC, β=0.310, p= 0.030; VMPFC, β=0.265, p=0.060; one-tailed permutation test).

Finally, the calculation of the pleasantness ratings still requires clarification. Each odor was presented 27–30 times, and if for each trial one of the 18 descriptors was selected, this would imply that pleasantness was rated only twice per odor before averaging. Is this correct? Additionally, were there odors for which only a single repetition was available?

We apologize for the lack of clarity. The pleasantness of each odor was rated at least 3 times. For S1 all three repetitions were inside the scanner (intensity and pleasantness were rated 3 times each in the scanner), while for S2 and S3 two ratings were acquired outside the scanner (all ratings acquired once inside the scanner). The pleasantness ratings were correlated across sessions in all subjects (Pearson's r: S1= 0.479, S2 = 0.668, S3 = 0.551, p<0.001, two-tailed t-test).

We have added this to Dataset description in the **Methods** section:

"The pleasantness of each odor was rated at least 3 times. For S1 all three ratings were collected inside the scanner, while for S2 and S3 two ratings were acquired outside the scanner. The pleasantness ratings were correlated across sessions in each subject (Pearson's r: S1= 0.479, S2 = 0.668, S3 = 0.551, p<0.001, two-tailed t-test)."

Reviewer #2 (Remarks to the Author):

In this revised manuscript, the authors have thoroughly addressed the comments provided before. A couple of minor clarifications would further enhance the readability of the manuscript text, as suggested below:

17: change from "these representations are separable in piriform cortex and amygdala" to "these pleasantness representations are separable for appetitive and aversive odors in piriform cortex and amygdala"

260: change from "importance" to "salience"

We thank the reviewer for re-reviewing our manuscript and we are glad that we have been able to adequately address the previous comments.

We have changed the flagged sentences accordingly.

Reviewer #3 (Remarks to the Author):

We thank the authors for their thoughtful responses to our concerns. Especially the in-depth reanalysis and control analyses for the issue raised concerning the voxelwise RSA are appreciated and address our concerns fully.
However, we still have a few concerns with the manuscript as it is. These relate to the following:

Point 1:
The additional explanations provided in the introduction insufficiently address my concerns about the terminology and theoretical framework used. I understand and appreciate the idea that pleasantness and unpleasantness likely represent two different dimensions, and the data presented by the authors beautifully supports this conclusion and the argument that the brain represents these dimensions separately.

My concern relates to the fact that the introduction represents the used operationalizations of valence and salience as orthogonal dimensions while one is in fact merely a absolute transformation of the other. This conversion, while arguably an appropriate descriptor for salience for a directly incentive-compatible stimulus like money, seems to be an oversimplification of the concept of salience in the case of odors. Given the various object identities and resulting associations that odors may have, multiple dimensions in addition to valence strength may contribute to their salience and one would expect that a separate measure over and above valence would be required to appropriately capture this, for example, a measure of memorability of the item in question such as a n-back task.

Also, given the interdependency between valence and salience as defined here, the authors' definition of salience is in disagreement with the affective neuroscience literature views on arousal and valence (where valence is truly independent of arousal, e. g. rage would be high negative valence and high arousal while sadness would be low valence and low arousal, and both might be salient), so I would argue that the authors' concept of salience does not overlap with arousal, as stated in the revision. I would recommend to remove this explanation.

I would like to reiterate that the finding of positive and negative valence as separable dimensions being represented through distinct cortical mechanisms is in itself a very interesting contribution. Particularly, the results of the cross-valence decoding analysis implies that pleasant and unpleasant stimuli are not represented using a common currency until the OFC. Framing this finding as separate coding of valence and salience in my opinion diminishes its impact.

We thank the reviewer for their comments. We agree that other perceptual features and attributes besides pleasantness may contribute to odor salience, which may be better captured by other

behavioral measures. We also agree that different sub-disciplines define and operationalize salience (or saliency) in different ways. For example, salience in vision sciences [1,2] is markedly different from uncertainty-based formulations in associative learning [3,4]. Given there is no definition of salience that is universally agreed upon[5], we feel that it is even more important to provide a clear operationalization of salience. We have therefore emphasized our operationalization, which is used in previous behavioral neuroscience and neuroeconomics studies[6-10], and clearly stated that other definitions (and multi-dimensional constructs) are possible. We also raise this issue in the discussion where we summarize alternate definitions and provide this as a limitation of our design.

We also fully agree that our definition of salience overlaps only very superficially with arousal, and have therefore revised the introduction and removed the link between salience and arousal.

Finally, we appreciate and fully agree with the comment emphasizing that our main finding is that positive and negative domains of pleasantness are represented through different cortical mechanisms.

Introduction

*Psychological theories often characterize affective experiences along continuous dimensions such as valence, intensity, salience and arousal[11-14]. Of note, different disciplines and studies define salience in different ways, emphasizing attention[1,2], uncertainty[3,4] or motivation and learning[5,6]. Here we adopt a framework from behavioral and decision neuroscience, in which stimulus pleasantness (more generally, stimulus value) gives rise to two continuous dimensions, namely valence (**Fig 1a** top) and salience (**Fig 1a** bottom). Whereas valence is based directly on pleasantness and thought to drive approach-avoidance responses [15,16], salience is defined as absolute pleasantness (very pleasant or very unpleasant odors have high salience) and relates to the magnitude of the affective response [7-10].*

Discussion:

We considered olfactory valence and salience as two candidate dimensions which could integrate across the distinct pleasantness representations. While valence (or value) is linearly related to pleasantness, salience has been defined in numerous ways across studies and disciplines [5]. For example, in vision sciences, salience or saliency relates to physical stimulus attributes that elicit attention[1,2], whereas definitions in the associative learning field focus on uncertainty and predictiveness [3,4]. Our definition is related to motivational salience in the behavioral neuroscience and neuroeconomics literature[6,10], operationalizing salience as the absolute value of pleasantness[7-9]. However, we do acknowledge that this formalization does not incorporate other potentially salience-related stimulus dimensions such as familiarity, intensity, arousal etc., which require additional ratings or behavioral assessments to be captured appropriately.

Point 2:
Thank you for the explanation for the exclusion of session indicators in the design matrix. However, based on the documentation for GLMsingle, multiple sessions should be accounted for in the sessionindicator input (c.f.

https://glmsingle.readthedocs.io/en/latest/wiki.html; **section ‘Things to watch out for when applying GLMsingle’, subsection ‘Data from multiple scan sessions’). This is due to the fact that response amplitudes can and often do vary across sessions. To my knowledge, this does not introduce them as regressors (and therefore would introduce problems of collinearity) but rather internally z-scores the responses within sessions and, according to the documentation, likely improves estimates of cross-validation performance. Note that the documentation uses the terminology where ‘sessions’ refer to separate scanning days/visits, in contrast to ‘runs’.**

We thank the reviewer for raising this subtle but important point. We acknowledge that GLMsingle documentation advises using session indicator functions or analyze each session separately. As a robustness check, we repeated our primary RSA and decoding analyses with session indicators included in GLMsingle. We observed little to no deviation from our previous results (Figure R1). This may be because our experiment comprised of a large number of trials distributed across 12 sessions and 160 odor conditions with pseudorandomization, which resulted in strong interleaving of conditions across sessions. In such a design, session-wise baseline shifts are expected to have less impact on beta estimates. Because of the robustness of our results to this parameter, we opted to stick with the original version of our analysis.

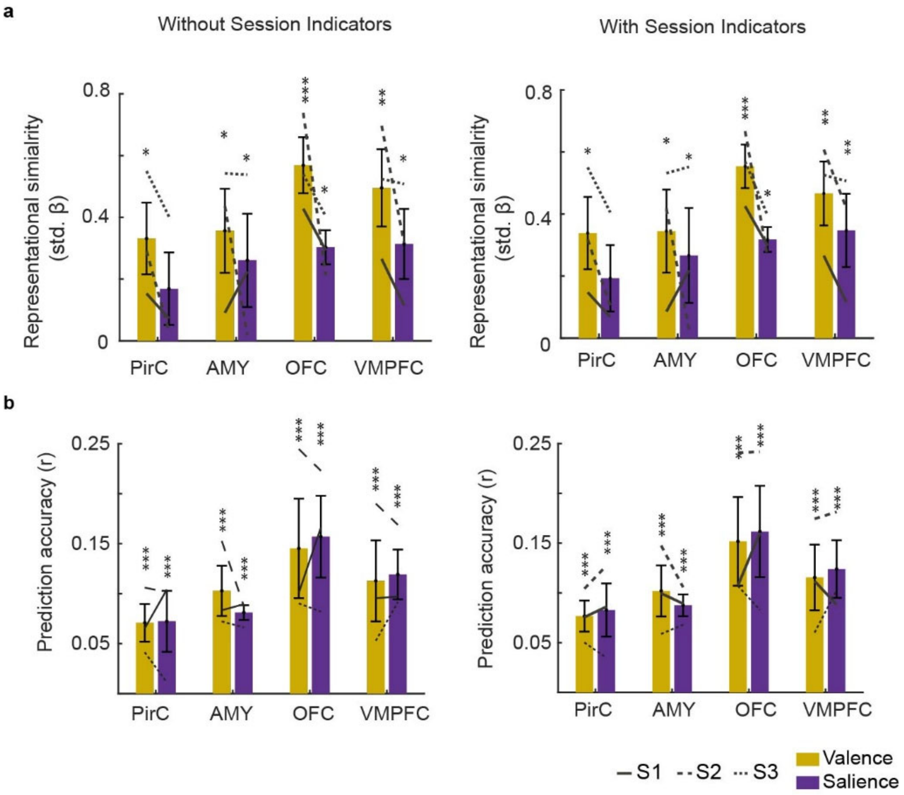

Review Figure R1. Representational similarity and decoding analyses without (left) and with session indicators (right). Left panels are the same as Fig 2c and 3b, for reference, Right panels show analyses after using GLM single with session indicators. **a left,** Standardized regression coefficients (beta estimates) for valence and salience from a linear model predicting the neural RSM. The coefficients for valence and salience are significant in all regions except salience for PirC. (valence: PirC, β=0.331, p=0.027; AMY, β=0.356, p=0.031; OFC, β=0.569, p<0.001; VMPFC, β=0.495,

p=0.001; salience: PirC, β=0.169, p=0.182; AMY, β=0.260, p=0.046; OFC, β=0.303, p=0.026; VMPFC, β=0.314, p=0.019; one-tailed permutation test)**; right,** Standardized regression coefficients (beta estimates) for valence and salience from a linear model predicting the neural RSM (corrected single trial estimates using session-wise indicators). The coefficients for valence and salience are significant in all regions except salience for PirC. (valence: PirC, β=0.338, p=0.027; AMY, β=0.345, p=0.034; OFC, β=0.553, p=0.001; VMPFC, β=0.466, p=0.0013; salience: PirC, β=0.192, p=0.141; AMY, β=0.266, p=0.037; OFC, β=0.318, p=0.012; VMPFC, β=0.3474, p=0.008; one-tailed permutation test). **b. left,** Prediction accuracy (Pearson's correlation between predicted and actual valence/salience in held out test sets) is significant in all brain regions. (valence: PirC, r=0.071, p<0.001; AMY, r=0.103, p<0.001; OFC, r=0.145, p<0.001; VMPFC, r=0.113, p<0.001; salience: PirC, r=0.072, p<0.001; AMY, r=0.081, p<0.001; OFC, r=0.157, p<0.001; VMPFC, r=0.119, p<0.001; N = 4400; two-tailed t-test). Data are presented as mean values and error bars indicate standard error of the mean. **right**, Prediction accuracy (Pearson's correlation between predicted and actual valence/salience in held out test sets, using corrected single trial estimates using session-wise indicators) is significant in all brain regions. (valence: PirC, r=0.076, p<0.001; AMY, r=0.102, p<0.001; OFC, r=0.151, p<0.001; VMPFC, r=0.115, p<0.001; salience: PirC, r=0.082, p<0.001; AMY, r=0.087, p<0.001; OFC, r=0.161, p<0.001; VMPFC, r=0.124, p<0.001; N = 4400; two-tailed t-test).

Point 3:
While reviewing the details concerning the methods above, I noticed that more details in the methods section of the manuscript itself would be very useful, and I would recommend that the authors consider adding those to the main manuscript in addition to their deposition of code and data to the repository. Key details that are missing are the software used, SVR parameters (number of folds, kernel, cost parameter etc.). There are also some minor inconsistencies that would benefit from further elaboration, for example the readme on the github repository says MATLAB 2023b with SPM12 and GLMsingle as necessary toolboxes, although the wrapper functions call on the svmtrain and svmpredict functions that have not been in use since MATLAB 2019 (outside of the LIBSVM toolbox).

Thank you for this suggestion – we have included these details in the methods section. Specifically, svmtrain and svmpredict are LibSVM functions that have the name as matlab's obsolete functions. We have added LibSVM as a toolbox in the github repository as well.

Methods:
For all decoding analyses, we chose the LibSVM SVR with default parameters (cost = 1, nu = 0.5, gamma = 1/num features) with radial kernel and split-half for cross validation.

Code Availability: *All analyses were performed using custom scripts in MATLAB 2023b and the LibSVM package[17] for decoding. Other software packages used in the study were SPM12[18], GLMSingle[19,20], and Breathmetrics toolbox[21].*

Finally, as a very minor point–the methods section mentions that Fisher's Z-transform was applied to the SVR results. If so, are the values reported on the figures the raw correlations or the z-transformed correlations?

These values reflect raw correlations. We have now clarified this in the manuscript.
Methods:

The values reported in the figures were inverse-transformed to reflect raw correlations.

Reviewer #4 (Remarks to the Author):

I co-reviewed this manuscript with one of the reviewers who provided the listed reports. This is part of the Nature Communications initiative to facilitate training in peer review and to provide appropriate recognition for Early Career Researchers who co-review manuscripts

Thank you for your comments and contribution.

References

1 Itti, L. & Koch, C. A saliency-based search mechanism for overt and covert shifts of visual attention. *Vision research* **40**, 1489-1506 (2000).

2 Fecteau, J. H. & Munoz, D. P. Salience, relevance, and firing: a priority map for target selection. *Trends in cognitive sciences* **10**, 382-390 (2006).

3 Cai, L. X. *et al.* Distinct signals in medial and lateral VTA dopamine neurons modulate fear extinction at different times. *Elife* **9**, e54936 (2020).

4 Esber, G. R. & Haselgrove, M. Reconciling the influence of predictiveness and uncertainty on stimulus salience: a model of attention in associative learning. *Proceedings of the Royal Society B: Biological Sciences* **278**, 2553-2561 (2011).

5 Bachman, M. D., Wang, L., Gamble, M. L. & Woldorff, M. G. Physical salience and value-driven salience operate through different neural mechanisms to enhance attentional selection. *Journal of Neuroscience* **40**, 5455-5464 (2020).

6 Berridge, K. C. & Robinson, T. E. What is the role of dopamine in reward: hedonic impact, reward learning, or incentive salience? *Brain research reviews* **28**, 309-369 (1998).

7 Kahnt, T., Park, S. Q., Haynes, J.-D. & Tobler, P. N. Disentangling neural representations of value and salience in the human brain. *Proceedings of the National Academy of Sciences* **111**, 5000-5005 (2014).

8 Bromberg-Martin, E. S., Matsumoto, M. & Hikosaka, O. Dopamine in motivational control: rewarding, aversive, and alerting. *Neuron* **68**, 815-834 (2010).

9 Matsumoto, M. & Hikosaka, O. Two types of dopamine neuron distinctly convey positive and negative motivational signals. *Nature* **459**, 837-841 (2009).

10 Lin, S.-C. & Nicolelis, M. A. Neuronal ensemble bursting in the basal forebrain encodes salience irrespective of valence. *Neuron* **59**, 138-149 (2008).

11 Russell, J. A. A circumplex model of affect. *Journal of personality and social psychology* **39**, 1161 (1980).

12 Kuppens, P., Tuerlinckx, F., Russell, J. A. & Barrett, L. F. The relation between valence and arousal in subjective experience. *Psychological bulletin* **139**, 917 (2013).

13 Winston, J. S., Gottfried, J. A., Kilner, J. M. & Dolan, R. J. Integrated neural representations of odor intensity and affective valence in human amygdala. *Journal of Neuroscience* **25**, 8903-8907 (2005).

14 Anderson, A. K. *et al.* Dissociated neural representations of intensity and valence in human olfaction. *Nature neuroscience* **6**, 196-202 (2003).

15 Yeshurun, Y. & Sobel, N. An odor is not worth a thousand words: from multidimensional odors to unidimensional odor objects. *Annual review of psychology* **61**, 219-241 (2010).

16 Jin, J., Zelano, C., Gottfried, J. A. & Mohanty, A. Human amygdala represents the complete spectrum of subjective valence. *Journal of Neuroscience* **35**, 15145-15156 (2015).

17 Chang, C.-C. & Lin, C.-J. LIBSVM: A library for support vector machines. *ACM transactions on intelligent systems and technology (TIST)* **2**, 1-27 (2011).

18 Ashburner, J. *et al.* SPM12 manual. *Wellcome Trust Centre for Neuroimaging, London, UK* **2464**, 53 (2014).

19 Allen, E. J. *et al.* A massive 7T fMRI dataset to bridge cognitive neuroscience and artificial intelligence. *Nature neuroscience* **25**, 116-126 (2022).
20 Prince, J. S. *et al.* Improving the accuracy of single-trial fMRI response estimates using GLMsingle. *Elife* **11**, e77599 (2022).
21 Noto, T., Zhou, G., Schuele, S., Templer, J. & Zelano, C. Automated analysis of breathing waveforms using BreathMetrics: a respiratory signal processing toolbox. *Chemical senses* **43**, 583-597 (2018).

RESPONSE TO REVIEWERS' COMMENTS

Reviewer #1 (Remarks to the Author):

In the revised manuscript, the authors have thoroughly addressed all my comments.

Reviewer #3 (Remarks to the Author):

We would like to thank the authors for the thoughtful considerations of our concerns, which have been fully addressed in this revision.

Reviewer #4 (Remarks to the Author):

I co-reviewed this manuscript with one of the reviewers who provided the listed reports. This is part of the Nature Communications initiative to facilitate training in peer review and to provide appropriate recognition for Early Career Researchers who co-review manuscripts.

Response:

We thank all the reviewers for re-reviewing our manuscript and we are glad that we have been able to adequately address the previous concerns.
